# Insights into the evolution of sorbitol metabolism: phylogenetic analysis of SDR196C family

**DOI:** 10.1186/1471-2148-12-147

**Published:** 2012-08-16

**Authors:** Agustín Sola-Carvajal, María I García-García, Francisco García-Carmona, Álvaro Sánchez-Ferrer

**Affiliations:** 1 Department of Biochemistry and Molecular Biology-A, Faculty of Biology, Regional Campus of International Excellence “Campus Mare Nostrum”, University of Murcia Campus Espinardo, Murcia, E-30100, Spain

## Abstract

**Background:**

Short chain dehydrogenases/reductases (SDR) are NAD(P)(H)-dependent oxidoreductases with a highly conserved 3D structure and of an early origin, which has allowed them to diverge into several families and enzymatic activities. The SDR196C family (http://www.sdr-enzymes.org) groups bacterial sorbitol dehydrogenases (SDH), which are of great industrial interest. In this study, we examine the phylogenetic relationship between the members of this family, and based on the findings and some sequence conserved blocks, a new and a more accurate classification is proposed.

**Results:**

The distribution of the 66 bacterial SDH species analyzed was limited to Gram-negative bacteria. Six different bacterial families were found, encompassing α-, β- and γ-proteobacteria. This broad distribution in terms of bacteria and niches agrees with that of SDR, which are found in all forms of life. A cluster analysis of sorbitol dehydrogenase revealed different types of gene organization, although with a common pattern in which the SDH gene is surrounded by sugar ABC transporter proteins, another SDR, a kinase, and several gene regulators.

According to the obtained trees, six different lineages and three sublineages can be discerned. The phylogenetic analysis also suggested two different origins for SDH in β-proteobacteria and four origins for γ-proteobacteria.

Finally, this subdivision was further confirmed by the differences observed in the sequence of the conserved blocks described for SDR and some specific blocks of SDH, and by a functional divergence analysis, which made it possible to establish new consensus sequences and specific fingerprints for the lineages and sub lineages.

**Conclusion:**

SDH distribution agrees with that observed for SDR, indicating the importance of the polyol metabolism, as an alternative source of carbon and energy. The phylogenetic analysis pointed to six clearly defined lineages and three sub lineages, and great variability in the origin of this gene, despite its well conserved 3D structure. This suggests that SDH are very old and emerged early during the evolution. This study also opens up a new and more accurate classification of SDR196C family, introducing two numbers at the end of the family name, which indicate the lineage and the sublineage of each member, i.e, SDR196C6.3.

## Background

The short-chain dehydrogenase/reductase (SDR) superfamily consists of NAD(P)(H)-dependent oxidoreductases that are distinct from the medium-chain dehydrogenase/reductase (MDR) and aldo-keto reductase (AKR) superfamilies [1]. Our knowledge of these superfamilies initially emerged from observations made concerning alcohol dehydrogenases of *Drosophila* and mammalian liver, which were seen to be clearly different [2,3]. Insect and bacterial alcohol and polyol dehydrogenases initially received less attention, since these enzymes were found to be different, and were only considered to be of prokaryotic and lower eukaryotic origin [2,3]. However, the discovery of similarities between these latter enzymes and human or mammalian prostaglandin, hydroxyl-steroid and other dehydrogenases, changed that view dramatically [4-7]. In addition, in recent years, interest in SDR enzymes has increased, since they are useful in biotechnological and analytical processes. About 25% of all dehydrogenases belong to the SDR superfamily [8].

Common to all types of oxidoreductases is the occurrence of a Rossmann-fold dinucleotide cofactor-binding motif, which has been found to be one of the most common protein folds [9,10]. Among SDR, no high sequence identity between different members is observed (about 20–30%), but all of them display a highly similar 3D structure, typically folding into a simple one-domain architecture with distinct conserved motifs, including the cofactor binding site at the N-terminal, structure stabilizing motifs, the active center, catalysis-enhancing sites and the substrate binding site, located in the highly variable C-terminal region [1,11]. Such a degree of 3D structure conservation indicates that ancestral dehydrogenases existed within each SDR family, and after multiple events, these ancestral dehydrogenases gave rise to the present system of subfamilies and classes found within each family [1].

Given the early origin of SDR, the subsequent divergence has had time to become quite pronounced. Hundreds of SDR enzyme activities and their corresponding families have been detected. Based on the similar coenzyme-binding structure, their active-site relationship and repetitive patterns, five SDR superfamily types have been discerned from different data banks, named as “classical”, “extended”, “intermediate”, “divergent” and “complex” SDR enzymes [12]. This divergence also includes different enzymatic activities, most of them dehydrogenases or reductases, but also lyases and some isomerases. The active-site Tyr residue, assisted by adjacent Lys, Asn and Ser residues, has been found to fit to the basic reaction mechanism in most cases, but also to reflect acid–base catalysis and proton transfers [1]. Thus, SDR proteins not only have a very distant origin, including a viral representation [1,13], presumably from a time when virally-mediated lateral gene transfer commonly occurred [14], but also show a wide range of activities, involving half of all enzyme activity types. Few gene/protein superfamilies exhibit this great divergence.

Recently, a sustainable and expandable nomenclature SDR database has been proposed, based on hidden Markov models (http://www.sdr-enzymes.org) [8,15]. This database has identified 314 SDR families, encompassing about 31,900 members [8]. Among them, the SDR196C family (http://www.sdr-enzymes.org/search) groups bacterial sorbitol dehydrogenases (L-iditol NAD + oxidoreductases, EC 1.1.1.14, SDH), which are of industrial interest for the specific determination of sorbitol (D-glucitol), a natural acyclic polyol found in food, and in pharmaceutical and cosmetic preparations [16].

In this study, we provide a comprehensive insight into the distribution, diversity, evolution and classification of the SDR196C superfamily in bacteria. The phylogenetic analysis revealed different lineages related to some sequence differences in the conserved SDR motifs and in the characteristic SDH blocks, allowing, for the first time, the classification of this SDR family (SDH family) into 6 different lineages and three sub lineages. This could permit a more efficient data curation, and a new nomenclature for the classification of incoming sequences into the SDR196C family.

## Results and discussion

### Distribution of SDH gene

The SDR database (http://www.sdr-enzymes.org) is a sustainable and expandable nomenclature database [8,15], which includes 127 bacterial sorbitol dehydrogenases (among characterized and putative) within the SDR196C family. Some identical sequences have been included two or more times in the database, representing different strains. In order to simplify the study, only one strain of these species was included in the analysis. The SDH gene was found in 66 bacterial species, all of them Gram-negative belonging to alpha (α)-, beta (β)- and gamma (γ)-Proteobacteria (see Additional file 1), with both low and high GC representatives (see Additional file 2). This distribution of SDH agrees with the prevalence of SDR enzymes, which are present in all forms of life [17] and, according to recent data from random genome screenings of microorganisms and viruses from sea water, are also among the most abundant genes in nature [13].

α-Proteobacteria, with six families, is the largest group with a total of 41 species, including members of the Acetobacteriace, Rhodospirillaceae, Rhodobacteriaceae, Brucellaceae, Phyllobacteriaceae and Rhizobiaceae families (see Additional file 1). Two of these species, *Ochrobactrum anthropi* and *O. intermedium* (members of the Brucellaceae family) are human pathogens that cause septicemia [18,19]. The Acetobacteriaceae family is represented by two members, *Acidiphilium cryptum* and *Gluconoacetobacter hansenii,* which are common in vinegar and used as iron contamination indicators [20]. Members of the Rhodospirilaceae, Phyllobacteriaceae and Rhizobiaceae families are usually nitrogen-fixing microorganisms found in soil and aquatic habitats, with the exception of *Agrobacterium tumefaciensA. radiobacter* and *A. vitis* from the Rhizobiaceae family, which are well known plant pathogens, causing tumors. Rhodobacteriaceae is the most numerous family, with 22 members, including sea water microorganisms, photosynthetic bacteria (*Rhodobacter* sp., *R. capsulatus* and *R. sphaeroides*[21]) and two extremophiles (*Paracoccus denitrificans* and *Silicibacter lacuscaerulensis)*.

The β-Proteobacteria group includes 15 members of the Burkholderiaceae family and 2 of the Commamonadaceae family (see Additional file 1). These two families are composed of soil and free-living microorganisms, which are usually nitrogen-fixing (*Burkholderia phymatum* and *B. xenovorans*) or symbionts (*B. graminisB. phytofirmansB. thailandensisAcidovorax avenae* and *Variovorax paradoxus*). The Burkholderiaceae family also includes a commensal of the earthworm nephridia (*Verminephrobacter eiseniae)*, a plant pathogen (*Ralstonia solanacearum*) and human pathogens from genera *Burkholderia,* which cause opportunistic infections in diseases, such as cystic fibrosis [22].

The γ-Proteobacteria group, which includes only 7 species from three different bacterial families, is the smallest group among the SDR196C family. Aquatic bacteria from the halophilic family Halomonadaceae (*Chromohalobacter salexigensis* and *Halomonas elongata*) and from the marine family Oceanospirillaceae (*Marinomonas* sp.) form part of this group. The third family of this group (Pseudomonaceae) includes a plant commensal (*Pseudomonas fluorescens)*, a plant pathogen (*P. savastanoi)* which cause olive knot [23], a saprophyte (*P. syringae),* and a soil bacterium (*Pseudomonas* sp.). Taken together, these results indicate that the SDH gene is widely distributed in nature, and is an important enzyme in the polyol metabolism of some bacteria, to use sorbitol as an alternative source of carbon and energy [21].

### Genetic organization of SDH gene

The three main bacterial groups that contain the SDH gene, α-, β- and γ-proteobacteria, encode this gene in different polyol clusters with a different gene order (see Additional file 3). In general terms, the SDH gene in the polyol operon is usually surrounded by a transporter (mainly, ATP Binding Cassette [ABC] transporter, which translocate substrates across membrane *via* ATP hydrolysis), an SDR protein (normally, a mannitol dehydrogenase, MDH) and a sugar related kinase, such as ribitol kinase. However, the companion genes and the order in the cluster, vary between bacterial families, and, to a lesser extent, within families (see Additional file 3). Only one overall organization of SDH genes has been described for the polyol operon of *Rhodobacter sphaeroides* Si4, where *smoS* gene encodes for an SDH, *smoK* for an ABC transporter, and *mtlK* for a mannitol dehydrogenase [24].

Within the α-Proteobacteria, there are twelve variants of the SDH cluster, each family having its own gene order. The Rhodobacteriaceae family shows three variants of this polyol operon, in which a cluster of four genes related with ABC transporter are on one side of the SDH gene, and a dehydrogenase (mannitol or alcohol dehydrogenase) plus an extra gene (HAD, tRNA or FeoA protein) on the other side (see Additional file 3, variants 1–3). The Rhizobiaceae family has more diversity in its polyol cluster, which displays 5 different variants (see Additional file 3, variants 4–8), but still shows the pattern of at least three ABC genes on one side of the SDH gene, except for *Agrobacterium tumefaciens* and *Rhizobium etli*, in which two ABC genes are replaced by two sugar kinase genes (fructose kinase and tagatose 6-phosphate kinase) (see Additional file 3, variant 6). On the other side of the SDH gene, a dehydrogenase (MDH or a Zn^2+^ binding dehydrogenase) is also present, except for *Agrobacterium radiobacter*, which presents an *AraC* regulator, followed by metal-accepting chemotaxis sensory transducer MACST (see Additional file 3, variant 5). This microorganism also has a *LysR* gene, indicating a tight regulation of the SDH related genes in order to use this sugar and to control its metabolism under adverse conditions. This control is also seen in *A. tumefaciens* and *R. etli* (see Additional file 3, variant 6), with the presence of *LacI*.

The rest of the α-Proteobacteria families (Phyllobacteriaceae, Rhodospirillaceae, Acetobacteriaceae and Brucellaceae) have the common pattern of at least three ABC proteins on one side, but are more diverse on the other side, having not only kinases (hexokinase or fructose kinase) but also two singular enzymes in SDH clusters, which are related with phosphogluconate (2-dehydro-3-deoxyphosphogluconate aldolase and phosphogluconate dehydrogenase) (see Additional file 3, variant 11), or two consecutive dehydrogenases (mannitol dehydrogenase and alcohol dehydrogenase; see Additional file 3, variant 12). The *LysR* gene is also present, except in the Rhodospirillaceae family.

Among the β-proteobacteria group, the Commamonadaceae family has its own order, but with the presence of an intercalating MDH gene between SDH and ABC transporter genes (see Additional file 3, variant 13). The latter order is also similar to that of *Verminephrobacter eiseniae,* a member of the Burkholderiaceae family (Additional file 3, variant 14), which is in fact, quite different from the common pattern displayed for the rest of the Burkholderiaceae members (see Additional file 3, variants 15–17). The latter clusters show a ferric uptake regulator gene and its corresponding cation ABC transporter genes on one side, and sorbitol/mannitol ABC transport genes followed by HAD gene on the SDH gene side (see Additional file 3, variant 15), and sometimes interrupted by two sugar related genes (ribokinase and tagatose 1,6-biphosphate aldolase, variant 16; or 2-keto-3-deoxygluconate kinase and tagatose 6-phosphate kinase, see Additional file 3, variant 17).

Finally, the γ-Proteobacteria group has no specific pattern, and it is easy to differentiate the Halomonadaceae family (see Additional file 3, variants 18–19), with an haloacid dehalogenase gene intercalating the SDH and three ABC transporter proteins, from the Pseudomonadaceae family (see Additional file 3, variants 20–21), in which only one (or no) ABC transporter gene is present, together with an *AraC* gene. This latter family also lacks the second SDR gene, indicating that the SDH gene is not close to other the sugar-utilizing genes as it is in all of the above described families.

### Signatures of horizontal gene transfer

Basically, there are two main methods to identify putative horizontal gene transfer events, phylogenetic methods and surrogate methods based on nucleotide composition. Also, the presence of transposases and/or integrases within a region may suggest another mode of transfer. However, no such enzymes genes were found in the proximity of any of the polyol clusters described above. On the other hand, the differences between the average GC content of whole genome (GC_g_) and the GC content of the SDH genes (GC_SDH_) (see Additional file 2), showed only one unknown possible horizontal gene transfer event (deviation from GC_g_ by +/− 5) in *Gluconoacetobacter hansenii* with a GC difference of −5.8 (see Additional file 2). In addition, *Loktanella vestfoldensis*, *Agrobacterium radiobacter*, *Chromohalobacter salexigensis*, *Pseudomonas savastanoi* and *Oceanicola batsensis* have a high, but not significant, GC difference (≈ +/− 4), which suggest the possibility of horizontal gene transfer for SDH in these species, too.

### Phylogenetic analysis of SDH gene

**Figure 1:**
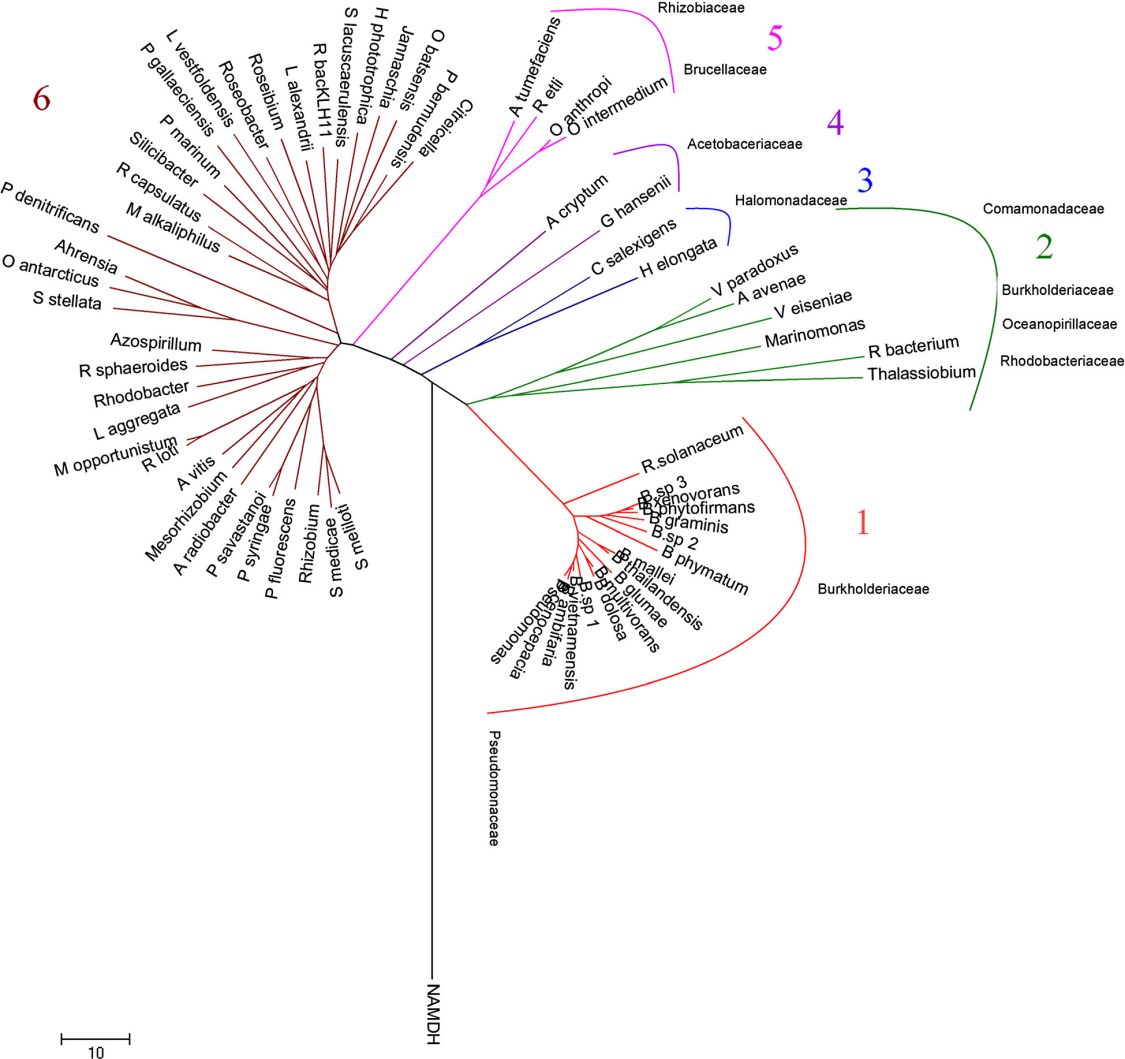
**Phylogenetic tree of all species encoding SDH gene.** The tree was obtained using Neighbor Joining (NJ) analysis in MEGA 4.0 [45]. 1,000 generations were used to build the consensus tree, as indicated in the methods section. The main inclusive taxonomic groups are indicated. The bacteria used are listed in Additional file 1.

**Figure 2:**
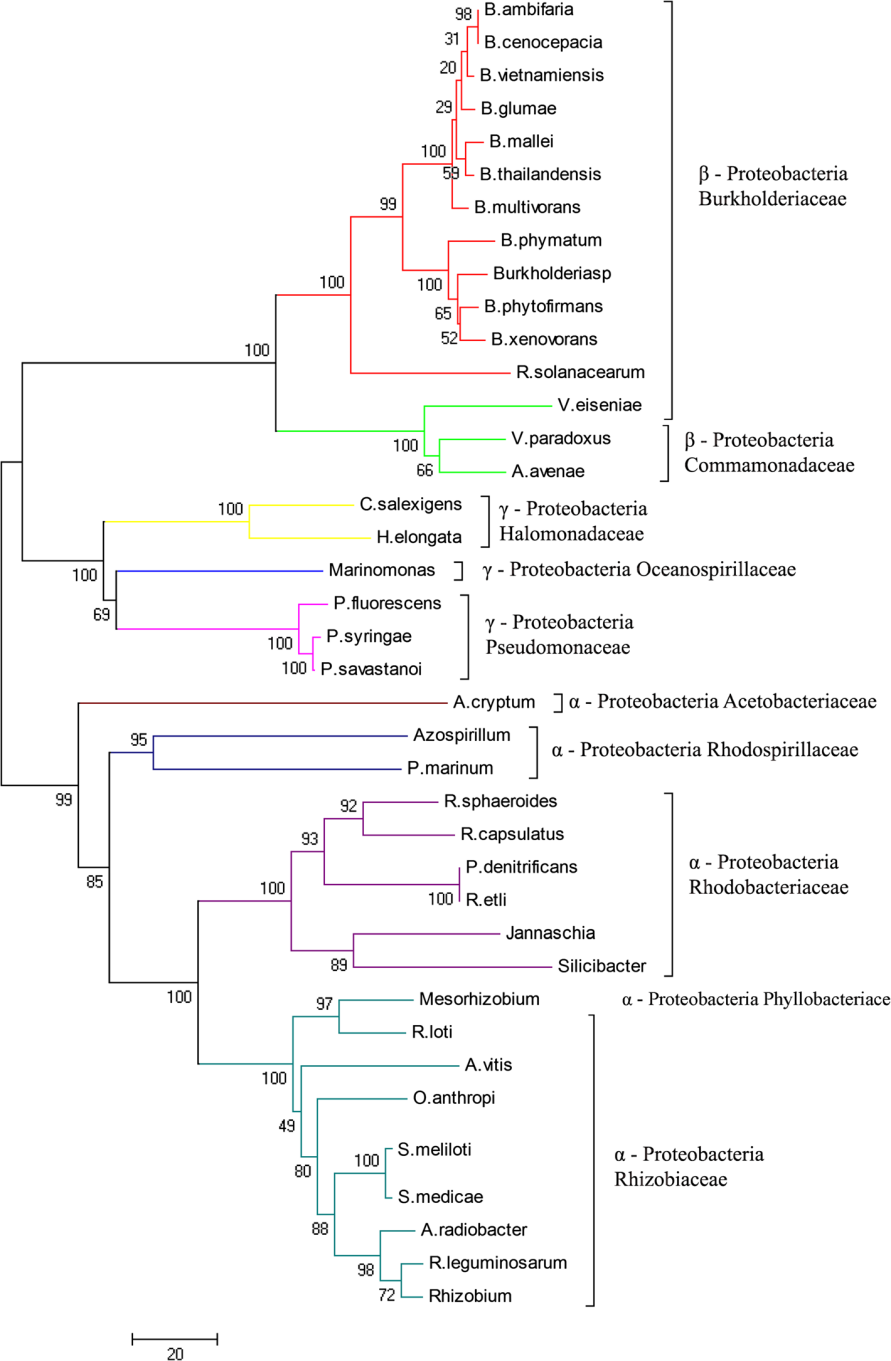
**Phylogenetic tree of 16S rRNA of all families encoding SDH gene.** Sequences for the analysis were obtained from GeneBank. The analysis was performed using the Neighbor Joining method, and the tree was implemented with MEGA 4.0 [45] after 1,000 generations. Families encoding SDH gene are indicated.

**Figure 3:**
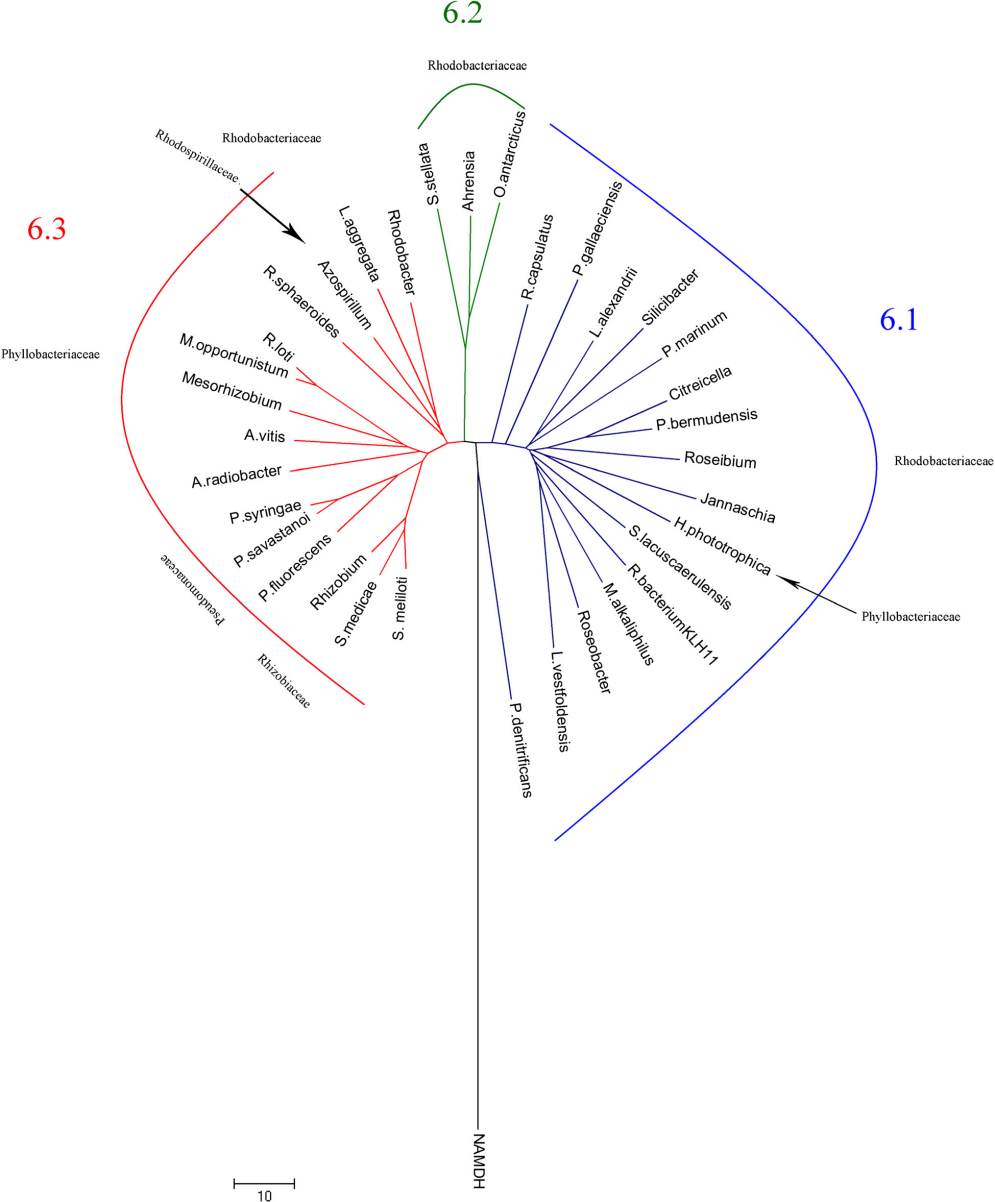
**Phylogenetic tree of species forming lineage 6 of SDH.** The tree was obtained using Neighbor Joining (NJ) analysis in MEGA 4.0 [45]. 1,000 generations were used to build the consensus tree, as indicated in the methods section. Main inclusive taxonomic groups are indicated.

In order to examine further the evolutionary history of the SDH gene, a multiple sequence alignment (MSA) was carried out with GUIDANCE [25,26], using the MSA algorithm PRANK [27], which gave an overall quality assessment exceeding 0.97 (1 corresponds to 100% certainty) (see Additional file 4). The phylogenetic analysis and the topology obtained were compared with that found for the species tree based on 16S rRNA sequences aligned with the above algorithm (Figure 1 and 2, respectively; see also Additional file 4). Phylogenetic analyses of SDH amino acid sequences resulted in a well-resolved tree, which was quite similar, regardless of the method used (see Additional file 5). Overall, the SDH genes in the three proteobacteria groups studied did not form three distinct lineages (Figure 1) as it does, in the 16S rRNA tree (Figure 2, see also Additional file 6). Indeed, the SDH tree could be subdivided into six main lineages (named 1, 2, 3, 4, 5 and 6) (Figure 1), lineage 6 being the most divergent, encompassing α- and γ-proteobacteria from five different families (Figure 1). Also within this lineage, three sublineages (Figure 3, see also Additional file 4) were found, all with a common origin. Lineages 6.1 and 6.2 were formed by species of the Rhodobacteriaceae family, except *Hoeflea phototrophobica*, which is member of the Phyllobacteriaceae family. Lineage 6.3 was the most divergent group, with members of α- and γ-proteobacteria from five different families: Phyllobacteriaceae, Pseudomonaceae, Rhodobacteriaceae, Rhodospirillaceae and Rhizobiaceae (Figure 3). This lineage 6 is also grouped the most SDH from the Rhodobacteriaceae family, except *R. bacterium* and *Thalassiobium* sp. Interestingly, the three members of genus *Rhodobacter* did not group as closely as might be expected, *R. capsulatus* being a member of lineage 6.1 and *R. sphaeroides* and *Rhodobacter* sp. members of lineage 6.3. This indicates a common ancestor, with a divergence in the time of SDH gene acquisition. Similarly, Pseudomonaceae members were grouped in lineage 6, except for *Pseudomonas* sp., which belongs to lineage 1, together with all members of Burkholderiaceae family (Figure 1 and 3). The distribution of the SDH from different *Pseudomonas* species in divergent branches of the tree (Figure 1) indicates that in these species, the SDH genes were acquired many times and from different sources. 

Lineage 1 contained most of the members of β-Proteobacteria family, except for the Commamonadaceae representatives, which branches away in lineage 2. In addition, lineage 1 branches firmly away from the rest of the lineages, suggesting that the origin of SDH in lineage 1 is unique, and that there are two different origins for β-proteobacteria SDH. Lineage 2 is also a divergent group which includes, apart from Commamonadaceae SDH proteins, members of α- and γ-Proteobacteria from the Rhodobacteriaceae and Oceanospirillaceae families. The presence of members of the Rhodobacteriaceae family (*Rhodobacterales bacterium* and *Thalassiobium* sp) clearly separated from lineage 6, indicated the possibility of a horizontal gene transfer event, although this is not supported by the GC difference, with values of −1.6 and 2.6, respectively, or by the presence of transposases/integrases within its polyol cluster.

Lineages 3, 4 and 5 are well separated groups, of different but close origin. Members of Halomonadaceae (lineage 3), Acetobacteriaceae (lineage 4), Brucellaceae and Rhizobiaceae (lineage 5) families are included in these lineages. *Agrobacterium tumefaciens* and *Rhizobium etli* did not group with the rest of their family in lineage 6, indicating a divergent origin of SDH in this family. Interestingly, γ-proteobacteria had at least one member in four of the six lineages described (Lineages 1, 2, 3 and 6), which suggests a divergent origin of the SDH gene among γ-proteobacteria.

This widespread and variable origin of SDH detailed here is related to the distribution and evolution of SDR, which were mentioned above, occurs in all kingdoms of life [17]. However, this variability is not observed in the structure of these enzymes in all six lineages (see Additional file 7), which all share a common Rossmann-fold motif for dinucleotide cofactor binding, and a substrate binding site in the highly variable C-terminal region [1]. This variability in the distribution and the homogeneity in the structure, together with the recombinatorial formation of the catalytic subunit from building blocks, suggest that SDR, and consequently SDH, emerged early from α/β elements to a form a Rossman-fold domain in the universal cellular ancestor prior to Darwinian evolution in cells of all kingdoms of life [17,28].

### Sequence comparison of SDH lineages

**Figure 4:**
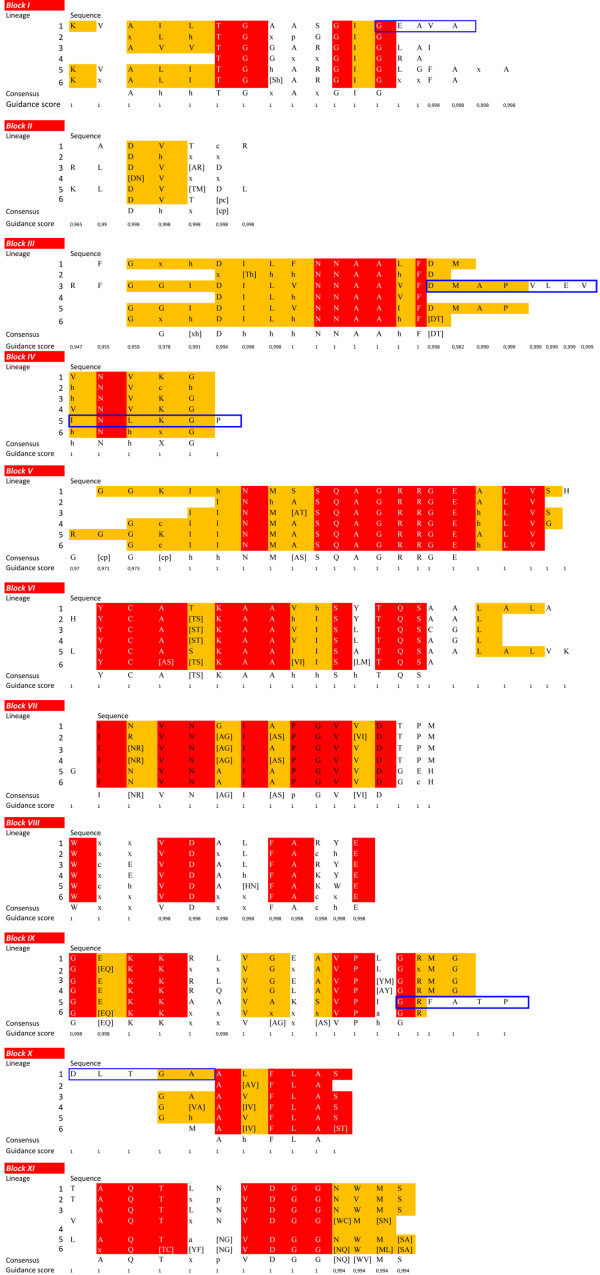
**Conserved SDH blocks in the six different lineages.** “c”, a charged residue; “h”, a hydrophobic residue; “p”, a polar residue and “x”, any residue. Alternative amino acids at a given position are shown within brackets. Red background indicates strictly conserved amino acids, orange background indicates conserved amino acids and blue boxes indicate the specific blocks of each lineage. Guidance scores represent the degree of confidently aligned residues (1 corresponds to 100% certainty) [25,26].

**Figure 5:**
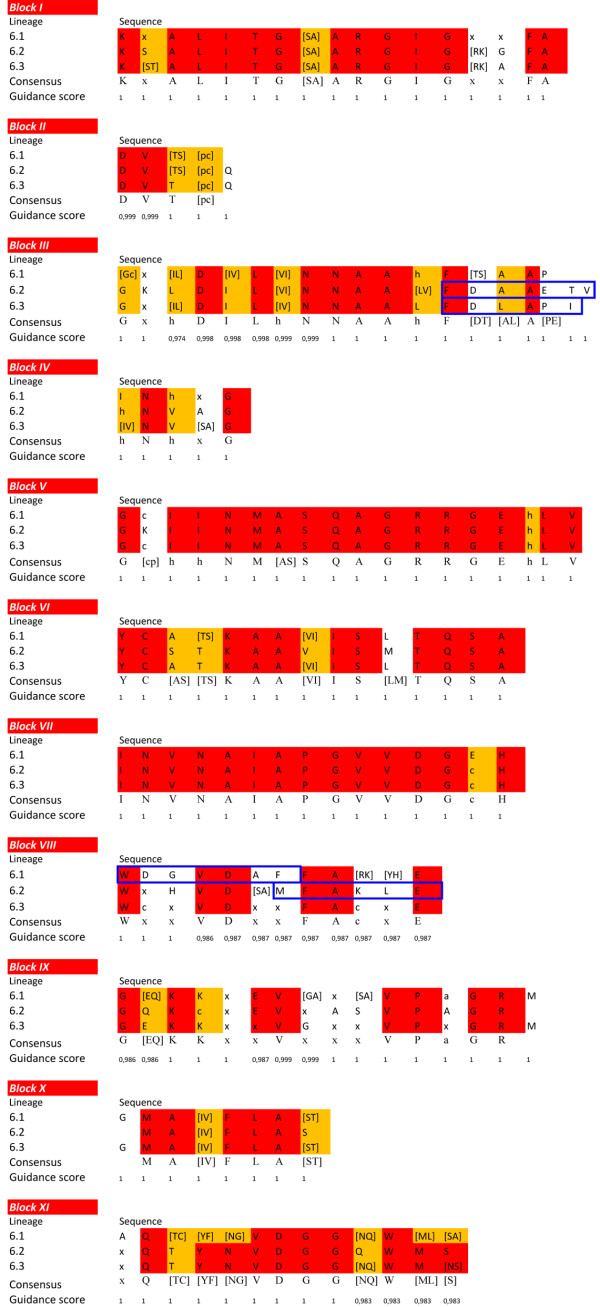
**Conserved SDH blocks in the three sublineages of lineage 6.** “c”, a charged residue; “h”, a hydrophobic residue; “p”, a polar residue and “x”, any residue. Alternative amino acids at a given position are shown within brackets. Red background indicates strictly conserved amino acids, orange background indicates conserved amino acids and blue boxes indicate the specific blocks of each lineage. Guidance scores represent the degree of confidently aligned residues (1 corresponds to 100% certainty) [25,26].

With the aim of further exploring the evolution of SDH proteins, 11 characteristic SDH blocks (I-XI) were described using the sequence alignment of the six different lineages and the 3 sublineages of lineage 6. Overall, differences in the blocks between the six lineages were well defined, and it was possible to establish different consensus sequences for each lineage with Guidance scores above 0.97 (Figure 4), which supports the classification obtained from the phylogenetic analysis. Lineages 2 and 6 were the most variable of the lineages (see Additional file 8), as might be expected according to the phylogenetic analysis (Figure 1), although it was still possible to establish consensus sequences. The eleven SDH conserved blocks were also observed in the three sublineages of lineage 6 with Guidance scores above 0.97 (Figure 5).

Sequence alignment of lineage 1 showed highly conserved blocks (see Additional file 8) among its members. This high degree of sequence similarity was in agreement with that described in the phylogenetic analysis, since lineage 1 was basically composed of members of the Burkholderiaceae family, except for *Pseudomonas* sp. The specific block sequence for this lineage is indicated in Figure 4, and, interestingly, the sequence of block I (GEAVA), which is involved in NAD^+^ binding [29-31] and the sequence of block X (DLTGA), which is related to NAD stabilization and tetramer formation, can be considered as fingerprints for this lineage, since these sequences were only present in this lineage. Lineage 2, as described above, was highly divergent, as also shown in the sequence alignment (see Additional file 8), where only the blocks corresponding to the characteristic fingerprints for SDH (block V to VII) and some parts of the C-ter are conserved. Block XI and its sequence NVMS could be considered as its fingerprint. Lineage 3, which comprises only two members, showed a high sequence identity (see Additional file 8). Its long and conserved block III, whose function is to stabilize the central β-sheet [29], could be considered as its fingerprint, ending in the conserved sequence DMAPVLEV (Figure 4). The most notable feature of lineage 4 alignment is the absence of block I in *G. hansenii*, which is involved in cofactor recognition (see Additional file 8). Taking this into account, its assignation as an SDH or even as an SDR might be erroneous, although the remaining conserved domains of both SDR and SDH, including the catalytic tetrad N-Y-S-K, responsible of the oxidoreductase activity [29], are present. Thus, the absence of block I is an error which arises from wrongly determining the starting point of sequence. Lineage 5 was also highly conserved (see Additional file 8), and showed the eleven blocks of SDH with high identity. Specific fingerprints of this lineage are located in block IV (INLKGP), and at the end of block IX, which in the other lineages usually ends with GRMG, while in lineage 5 it ends in FATP (Figure 4). The heterogeneity of lineage 6 makes it difficult to establish a fingerprint, except for the consensus sequence of block V (see Additional file 8). However, when the three sublineages were aligned independently, a high degree of conservation was observed (Figure 5). Lineage 6.1 was the most divergent, showing identity only in the eleven conserved blocks (see Additional file 8). Although consensus sequences are shown for every block, only a specific sequence for this sublineage was observed at the beginning of block VIII (WDGVDAF, Figure 5). Similarly, in lineage 6.2, specific fingerprints could be found in block III (FAAAETV), and at the end of block VIII (MFAKLE). Lineage 6.3 had its own fingerprint at the end of block III (FDLAPI).

In addition, some lineages also presented highly conserved sequences between conserved blocks. Thus, lineages 1, 3 and 5 showed such a sequence between motifs I and II (CVLVD, EAGRV and EGAcFcIADI, respectively), and another between block IV and V in lineages 1, 3, 4 and 5 (FFLMQAVA, FFTLQAVAA, QAVAxQMI, and FMMKAVSNVMI, respectively). These latter residues are part of the large α_5,_ which is part of the sorbitol binding domain in SDH. Such a degree of conservation and the 3D proximity to blocks IV and V, which are part of the active site, suggests a role for these interblock sequences in making up the correct structure of the active site or the substrate binding site.

To expand the above analysis, a study of SDH family functional divergence was carried out to detect amino acid sites that have varying evolutionary conservation among member genes, using DIVERGE (DetectIng Variability in Evolutionary Rates among Genes) software [32,33]. The analysis grouped the amino acids residues responsible for altered functional constraints into two categories: (I) conserved in the first lineage, but variable in the second lineage; (II) conserved in the second lineage, but variable in the first lineage. A site-specific profile based on probability (Q_k_) was used to identify critical amino acids [34], with a Q_k_ > 0.75 (see Additional file 9). Among the six lineages, only I, II, V and VI were relevant (see Additional file 10). In fact, when lineages I-II were compared, only 3 amino acids (IDD) were conserved in category II. Lineages I-V showed only one amino acid (R) in category I, and two (DR) in category II. The divergence was clearly more pronounced between lineage I and VI, with four amino acids (LPRE) in category I and 9 (IDAGIIAIG) in category II. This divergence pattern was also observed between lineages II and VI, with four amino acids (LDLD) in category I and 12 (FAIVIDAAGMRL) in category II. Finally, the divergence between lineage V and VI was reduced to only one amino acid (K) in category I.

**Figure 6:**
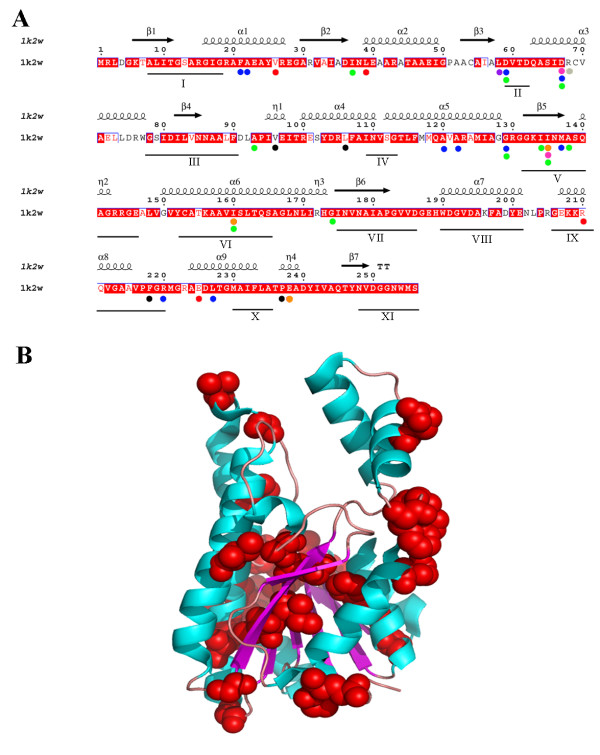
**The site-specific profile fro predicting critical amino acid residues for the functional divergence between SDH families.A**) Critical amino acid residues are represented by circles on the sequence of R*. sphaeroides* SDH (pdb: 1k2w). Symbols above the sequence represent the secondary structure, springs represent helices and arrows represent β-strands. Conserved blocks of bacterial SDH (I to XI) are indicated below the sequence alignment. Triangles represent the location of the active site. Circles represent the divergent amino acids according to the following color code: Grey; conserved amino acids in Lineage I and divergent in Lineage V, Red; conserved amino acids in Lineage I and divergent in Lineage VI, Orange; conserved amino acids in Lineage II and divergent in Lineage I, Black; conserved amino acids in Lineage II and divergent in Lineage VI, Pink; conserved amino acids in Lineage V and divergent in Lineage I, Purple; conserved amino acids in Lineage V and divergent in Lineage VI, Green; conserved amino acids in Lineage VI and divergent in Lineage I, Blue; conserved amino acids in Lineage VI and divergent in Lineage II. **B**) Representation of divergent amino acids in the structure of 1k2w. α-helices are indicated in cyan, β-sheets are indicated in purple, loops are indicated in light pink and the divergent amino acids as red spheres. Structure was rendered using PyMol [50].

To visualize these divergence sites, a 3D representation was carried out for each lineage (see Additional file 10) and for all sites together using *Rhodobacter sphaeroides* sequence and its crystal structure (pdb: 1k2w) [35] (Figure 6). At first sight, it was clear that divergent amino acids are basically outside the main conserved blocks, clearly indicating that the drift at these sites (shown by different colors in Figure 6A) is well tolerated by the structure with no loss of activity. The changes are outside block I (NADH-binding domain), block III (which stabilizes the central β-sheet), block VII (which determines the reaction direction) and blocks VIII and IX (involved in the cap domain, which defines the substrate channel, Figure 6B). However, as shown in Figure 6B, the main changes are located in and around β5, which contains one of the amino acids of the catalytic tetrad (S139, 1k2w numbering), which is not affected. Thus, it could be concluded that the sequence alignment of the different lineages obtained according to the phylogenetic and functional divergence analysis showed specific fingerprints for each lineage, whose conserved sequences could be very useful for the future classification of SDH enzymes. 

## Conclusions

SDR196C family encompasses short chain SDH of prokaryotic origin. The distribution of this family is limited to Gram-negative bacteria, grouping members of the α-, β- and γ-Proteobacteria. This distribution is in agreement with the widespread nature of SDR, and indicates that sorbitol metabolism is of importance among these bacteria as an alternative source of carbon and energy. This variability is also observed in the genetic organization of polyol operon among the different species, and in the phylogenetic analysis, which clearly point to different origins for SDH in bacteria, although the 3D structure among groups is highly conserved, with the typical Rossmann fold motif. Such a degree of divergence in the origin but similarity in the structure suggests that SDHs are of extremely old, and emerged early in the evolution, giving rise to the six different lineages and the three sublineages observed in the phylogenetic analysis. This phylogenetic classification is supported by the sequence differences found in the conserved blocks of SDH, allowing for the first time, the classification of the SDR196C family into different subgroups, and introducing the possibility of expanding the actual classification of SDR enzymes by two extra numbers, the lineage and sublineage, separated by a point. As an example, *R. capsulatus* SDH could be classified as SDR196C6.3.

## Methods

### Sequence retrieval and cluster identification

Protein sequences were obtained from the SDR-enzyme database (http://www.sdr-enzymes.org), a sustainable and expandable nomenclature database based on hidden Markov models [8,15]. The DNA sequences of the 16S rRNA from species encoding the SDH gene were from GenBank. When several strains from the same species encoded the same SDH gene, only one representative strain was included, the strain from the first sequenced genome.

### Sequence alignment

The sequences were aligned using GUIDANCE [25,26] with the MSA algorithm PRANK [27]. The alignments were further checked manually using Gene-Doc [36]. Large gaps and hyper variable sites were removed from the alignments; the same methodology was applied to gaps at the beginning and end of the alignment, which represent missing sequence data. Aligned sequences and their secondary structure are shown using ESPript [37].

### Phylogenetic analysis

Prot test and model test (protein and DNA sequences, respectively) were used in order to choose the most appropriate method to calculate the distances [38]. WAG with invariable sites for the SDH protein sequences and GTR with invariable sites for 16S rRNA sequences were used [39,40]. Three different tree-building methods were used: Maximum Likelihood (ML), Bayesian analysis (BY) and Neighbor Joining (NJ), as implemented in PHYML, MrBayes 3.1.2, and MEGA 4, respectively [41-43]. The bootstrap values for ML and NJ trees were obtained after 1,000 generations. For the trees constructed using BY, the Markov chains were run for 1,000,000 generations. The burn-in values were set for 10,000 generations, and the trees were sampled every 100 generations. Splitstree and MEGA 4 tree viewer were used to visualize the trees and calculate confidence values [44,45].

### Functional divergence analysis

Type I functional divergence was tested according to the previously described methods, using the DIVERGE software [33]. The alignment used for the phylogenetic analysis was also used for this application. The tree obtained by NJ was refined using the tool PROTTEST [46], to determine the best evolutionary model for the set of query proteins [47]. The crystal structure 1k2w was used to determine the location of divergent amino acids according to the analysis obtained. The test could not be applied to lineages III and IV, since DIVERGE needs at least 4 species to be considered a cluster. A site-specific profile based on probability (Q_k_) was used to identify critical amino acids [34], with a Q_k_ > 0.75. The values obtained for the critical amino acids and their location in the alignment according to DIVERGE are shown in Additional files 9,10,11, and 12.

### GC content

The GC content of the sequences was calculated and compared to the GC content of the whole genome. The formula used for the calculations was that described by Karlin *et al*., 2001 [48].

### Molecular modeling

Protein sequences were 3D modeled with Geno3D [49] and molecular representation were performed by Pymol [50].

## Abbreviations

SDR : Short-chain dehydrogenase/reductase(s); SDH : Sorbitol dehydrogenase(s); ABC : ATP-binding cassette; MDR : Medium-chain dehydrogenase/reductase(s); AKR : Aldo-keto reductase(s); HAD : Haloacid dehalogenase(s); MDH : Mannitol dehydrogenase(s); ML :Maximum likelihood; BY : Bayesian analysis; NJ : Neighbor joining.

## Competing interests

The authors declare that they have no competing interest.

## Authors’ contributions

ASF and FGC designed research. MIGG did sequence retrieval and alignments with ESPript. ASC carried out phylogenetic analysis by using related computer programs, and together with ASF, drafting of the manuscript. All authors read and approved the final manuscript.

## Supplementary Material

Additional file 1 **Distribution of SDH gene among bacteria.** The table indicates the bacterial species that encode a SDH gene, its taxonomy, ecology and niche. Click here for file

Additional file 2 **GC content differences between SDH genes and genome.** The table indicates the differences in GC content between the SDH gene and the core genome of the bacterium, where it is encoded. Click here for file

Additional file 3 **Structure of the SDH clusters among bacterial groups.** SDH gene was placed in the middle to facilitate cluster visualization. Variants of the cluster among taxonomic groups are represented by numbers (1–21). Click here for file

Additional file 4 **Multiple sequence alignments (MSAs) obtained with GUIDANCE and their corresponding Guidance scores [**[25,26]. The file shows the result obtained for the MSA of the 6 lineages, 3 sublineages and 16S rRNA. In all cases, an overall quality assessment (Guidance score) of above 0.97 was obtained with the MSA algorithm PRANK [27]. Click here for file

Additional file 5 **Phylogenetic trees of bacteria containing SDH gene.** The file shows two phylogenetic trees of SDH gene using Bayesian and Maximun Likelihood, as tree building methods. Click here for file

Additional file 6 **Phylogenetic trees (16S rRNA) of bacteria containing the SDH gene.** The file contained two phylogenetic trees of 16S rRNA of bacteria containing SDH gene, using Bayesian and Maximum Likelihood, as tree building methods. Click here for file

Additional file 7 **Molecular modeling of SDH enzymes from the six lineages described in bacterial SDH.** The proteins were modeled with Geno3D [49] and rendered by PyMol [50]. Selected proteins were Uniprot codes: lineage 1, A3MHB9; lineage 2, A1WMN; lineage 3 E1VCL7; lineage 4, A5FVQ; lineage 5, A9CES4; lineage 6.1, O68112; lineage 6.2, A3K129; lineage 6.3, A3PKH5. Click here for file

Additional file 8 **Multiple sequence alignment of bacterial SDH proteins.** ESPript [37] output was obtained with the aligned sequences from Additional file 1. Strictly conserved residues have a solid background. Symbols above sequences represent the secondary structure. Conserved structural blocks (I-XI) are shown below the sequences. Circles represent the divergent amino acids according to the following color code: Grey; conserved amino acids in Lineage I and divergent in Lineage V, Red; conserved amino acids in Lineage I and divergent in Lineage VI, Orange; conserved amino acids in Lineage II and divergent in Lineage I, Black; conserved amino acids in Lineage II and divergent in Lineage VI, Pink; conserved amino acids in Lineage V and divergent in Lineage I, Purple; conserved amino acids in Lineage V and divergent in Lineage VI, Green; conserved amino acids in Lineage VI and divergent in Lineage I, Blue; conserved amino acids in Lineage VI and divergent in Lineage II. Click here for file

Additional file 9 **Predicted critical amino acid sites responsible for functional divergence.** The table represents the values of Q_k_ of all pair analyses. Values with a Q_k_ > 0.75 are indicated with a red background. Click here for file

Additional file 10 **Candidates for amino acid sites related with functional divergence.** The numbering used corresponds with that of the alignment implemented by DIVERGE. Cat. I refers to Category I and Cat. II refers to Category II. A: Cat. II; conserved tandem in Lineage II and variable in Linage I. B: Cat. I; Conserved tandem in Lineage I and variable in Lineage V and Cat. II; conserved tandem in Lineage V and variable in Lineage I. C: Cat. I; conserved tandem in Lineage V and variable in Lineage VI. D: Cat. I; conserved tandem in Lineage I and variable in Lineage VI and Cat. II; conserved tandem in Lineage VI and variable in Lineage I. E: Cat. I; conserved tandem in Lineage II and variable in Lineage VI and Cat. II; conserved tandem in Lineage VI and variable in Lineage II. Critical amino acids responsible for functional divergence are shown in the *R. sphaeroides* SDH crystal structure (pdb:1k2w). Divergent residues of category I are depicted in red and those of category II are depicted in blue. Click here for file

Additional file 11 Alignment of SDH family in ClustalW format (.aln) used in the DIVERGE analysis. Click here for file

Additional file 12 Phylogenetic tree in PhylM (.ph) used in the DIVERGE analysis. Click here for file
