# Supplementary material for: Insights into the evolution of sorbitol metabolism: phylogenetic analysis of SDR196C family
Source: BMC Evol Biol. 2012 Aug 16;12:147. doi: 10.1186/1471-2148-12-147 (PMC3458964; doi:10.1186/1471-2148-12-147)
Supplement: Additional file 1 — Distribution of SDH gene among bacteria. The table indicates the bacterial species that encode a SDH gene, its taxonomy, ecology and niche. [file 1471-2148-12-147-S1.pdf]

Species	Uniprot ID	Taxonomy	Ecology	Niche/Disease
Puniceispirillum marinum	D5BSG2	α-prot	F	Sea water
Acidiphilium cryptum	A5FVQ6	α-prot Acet	F	Iron contaminated habitats
Gluconacetobacter hansenii ATCC 23769	D5QF12	α-prot Acet	F	Vinegar
Ochrobactrum anthropi	A6X5Q1	α-prot Brucell	P	Septicaemia
Ochrobactrum intermedium LMG 3301	C4WMK5	α-prot Brucell	P	Septicaemia
Hoeflea phototrophica DFL-43	A9DEK6	α-prot Phyll	F	Sea water
Mesorhizobium sp	Q11BS6	α-prot Phyll	F	S/Nitrogen fixing
Mesorhizobium opportunistum WSM2075	C8SIV9	α-prot Phyll	F	S/Simbiotic
Agrobacterium tumefaciens	A9CES4	α-prot Rhiz	F/PP	S/Tumors
Rhizobium etli	Q2K1R3	α-prot Rhiz	F	S/Simbiotic
Sinorhizobium meliloti BL225C	E0JJX4	α-prot Rhiz	F	Nitrogen fixing
Rhizobium loti	Q98D05	α-prot Rhiz	F	S/Nitrogen fixing
Sinorhizobium medicae	A6UC06	α-prot Rhiz	F	S/Nitrogen fixing
Agrobacterium radiobacter	B9JPZ8	α-prot Rhiz	F/PP	S/Tumors
Agrobacterium vitis	B9JRG2	α-prot Rhiz	F/PP	S/Tumors
Rhizobium sp	C3MGF5	α-prot Rhiz	F	S/Nitrogen fixing
Rhodobacterales bacterium HTCC2255	Q0FGE0	α-prot Rhod	F	Sea water
Thalassiobium sp	C7D981	α-prot Rhod	F	Sea water
Rhodobacter capsulatus	O68112	α-prot Rhod	F	Photosyntetic Bacteria
Phaeobacter gallaeciensis 2	A9EM40	α-prot Rhod	F	Sea water
Jannaschia sp	Q28N89	α-prot Rhod	F	Sea water
Paracoccus denitrificans	A1BBK7	α-prot Rhod	F	V/Extremophile
Silicibacter sp	Q1GJK6	α-prot Rhod	F	Sea water
Oceanicola batsensis HTCC2597	A3TT65	α-prot Rhod	F	Sea water
Loktanella vestfoldensis SKA53	A3V1Z9	α-prot Rhod	F	Sea water
Maritimibacter alkaliphilus HTCC2654	A3VGG9	α-prot Rhod	F	Sea water
Roseobacter sp	A4EEU1	α-prot Rhod	F	Sea water
Pelagibaca bermudensis HTCC2601	Q0FIL3	α-prot Rhod	F	Sea water
Rhodobacteraceae bacterium KLH11	B9NLC2	α-prot Rhod	F	Sea water
Labrenzia alexandrii DFL-11	B9R3J5	α-prot Rhod	F	Sea water
Silicibacter lacuscaerulensis ITI-1157	D0CP77	α-prot Rhod	F	Geothermal powerplant
Citreicella sp	D0D101	α-prot Rhod	F	Sea water
Roseibium sp	E2CFT5	α-prot Rhod	F	Sea water
Sagittula stellata E-37	A3K129	α-prot Rhod	F	Sea water
Octadecabacter antarcticus 307	B5J1N9	α-prot Rhod	F	Sea water
Ahrensia sp	E0MJ95	α-prot Rhod	F	Sea water
Rhodobacter sphaeroides	A3PKH5	α-prot Rhod	F	Photosyntetic Bacteria
Labrenzia aggregata IAM 12614	A0NY14	α-prot Rhod	F	Sea water
Rhodobacter sp	C8RZW7	α-prot Rhod	F	Photosyntetic Bacteria
Azospirillum sp	D3P2X3	α-prot Rhodosp	F	S/Nitrogen fixing
Burkholderia mallei	A3MHB9	β-prot Burkh	P	Glanders
Burkholderia ambifaria	B1YVT1	β-prot Burkh	F/P	V/Cystic Fibrosis
Burkholderia cenocepacia	Q1BU16	β-prot Burkh	F/P	V/Cystic Fibrosis
Burkholderia sp	Q39DE3	β-prot Burkh	F	Organic compounds
Burkholderia multivorans	A9AFX9	β-prot Burkh	F/P	V/Cystic Fibrosis
Ralstonia solanacearum	Q29SS8	β-prot Burkh	F/PP	S/Bacterial wilt
Burkholderia phymatum	B2JDG4	β-prot Burkh	F	Nitrogen fixing
Burkholderia phytofirmans	B2SYA3	β-prot Burkh	F	S
Burkholderia thailandensis	Q2T0Q1	β-prot Burkh	F	S
Burkholderia vietnamiensis	A4JHF0	β-prot Burkh	F	Paddies
Burkholderia xenovorans	Q13UM4	β-prot Burkh	F	S/Nitrogen fixing
Burkholderia dolosa AUO158	A2W7U7	β-prot Burkh	F/P	V/Cystic Fibrosis
Burkholderia graminis C4D1M	B1G234	β-prot Burkh	F	S
Burkholderia glumae	C5ACC3	β-prot Burkh	F/P	Oportunistic
Verminephrobacter eiseniae	A1WMN9	β-prot Burkh	C	Earthworms nephridia
Variovorax paradoxus	C5CPP3	β-prot Comamon	F	S
Acidovorax avenae subsp	D1STI6	β-prot Comamon	F	S
Chromohalobacter salexigens	Q1QZV8	γ-prot Halom	F	Salterns
Halomonas elongata DSM 2581	E1VCL7	γ-prot Halom	F	High salinity water
Marinomonas sp	A3YBC1	γ-prot Ocean	F	Sea water
Pseudomonas sp	Q5KTZ9	γ-prot Pseud	F	V
Pseudomonas syringae pv	Q88AM6	γ-prot Pseud	F/PP	Saprofit/various
Pseudomonas fluorescens	C3K8P4	γ-prot Pseud	C	Plant
Pseudomonas savastanoi pv	D7I663	γ-prot Pseud	PP	Olive knot

Taxonomy legend	
Burkh	Burkholderiaceae
Rhiz	Rhizobiaceae
Pseud	Pseudomonaceae
Rhod	Rhodobacteriaceae
Acet	Acetobacteriaceae
Halom	Halomonadaceae
Phyll	Phyllobacteriaceae
Brucell	Brucellaceae
Ocean	Oceanospirillales
Comamon	Comamonadaceae
Rhodosp	Rhodospirillaceae

Ecology legend	
P	Pathogen
F	Free living
PP	Plant Pathogen
C	Comensal

Niche/Disease legend	
V	Various
S	Soil
