# Supplementary material for: Insights into the evolution of sorbitol metabolism: phylogenetic analysis of SDR196C family
Source: BMC Evol Biol. 2012 Aug 16;12:147. doi: 10.1186/1471-2148-12-147 (PMC3458964; doi:10.1186/1471-2148-12-147)
Supplement: Additional file 2 — GC content differences between SDH genes and genome. The table indicates the differences in GC content between the SDH gene and the core genome of the bacterium, where it is encoded. [file 1471-2148-12-147-S2.pdf]

Additional File 2. Sola-Carvajal *et al*. 2011

Species	uniprot_ac	GC content difference
Acidiphilium cryptum	A5FVQ6	-0,7
Acidovorax avenae subsp	D1STI6	1,8
Agrobacterium radiobacter	B9JPZ8	-4,5
Agrobacterium tumefaciens	A9CES4	1,3
Agrobacterium vitis	B9JRG2	-1,2
Ahrensia sp	E0MJ95	-1,1
Azospirillum sp	D3P2X3	0,9
Burkholderia ambifaria	B1YVT1	2,6
Burkholderia cenocepacia	Q1BU16	1,7
Burkholderia dolosa AUO158	A2W7U7	2,9
Burkholderia glumae	C5ACC3	1,7
Burkholderia graminis C4D1M	B1G234	2,6
Burkholderia mallei	A3MHB9	0,5
Burkholderia multivorans	A9AFX9	2,2
Burkholderia phymatum	B2JDG4	1,4
Burkholderia phytofirmans	B2SYA3	0,7
Burkholderia sp	Q39DE3	3,2
Burkholderia thailandensis	Q2T0Q1	2,3
Burkholderia vietnamiensis	A4JHF0	3
Burkholderia xenovorans	Q13UM4	1,4
Chromohalobacter salexigens	Q1QZV8	4
Citreicella sp	D0D101	-0,3
Gluconacetobacter hansenii ATCC 23769	D5QF12	-5,8
Halomonas elongata DSM 2581	E1VCL7	0,4
Hoeflea phototrophica DFL-43	A9DEK6	0,9
Jannaschia sp	Q28N89	0,6
Labrenzia aggregata IAM 12614	A0NY14	3
Labrenzia alexandrii DFL-11	B9R3J5	2,4
Loktanella vestfoldensis SKA53	A3V1Z9	-4,7
Marinomonas sp	A3YBC1	1
Maritimibacter alkaliphilus HTCC2654	A3VGG9	
Mesorhizobium opportunistum WSM2075	C8SIV9	0,7
Mesorhizobium sp	Q11BS6	
Oceanicola batsensis HTCC2597	A3TT65	4,6
Ochrobactrum anthropi	A6X5Q1	0,2
Ochrobactrum intermedium LMG 3301	C4WMK5	-1,3
Octadecabacter antarcticus 307	B5J1N9	0,2
Paracoccus denitrificans	A1BBK7	1
Pelagibaca bermudensis HTCC2601	Q0FIL3	3,4
Phaeobacter gallaeciensis 2	A9EM40	1,1
Pseudomonas fluorescens	C3K8P4	3,1
Pseudomonas savastanoi pv	D7I663	4
Pseudomonas sp	Q5KTZ9	
Pseudomonas syringae pv	Q88AM6	1,9
Puniceispirillum marinum	D5BSG2	2,1
Ralstonia solanacearum	Q29SS8	-1
Rhizobium etli	Q2K1R3	-0,9
Rhizobium leguminosarum bv	Q1MLK9	1,9
Rhizobium loti	Q98D05	1
Rhizobium meliloti	Q92N06	1,8
Rhizobium sp	C3MGF5	0,8
Rhodobacter capsulatus	O68112	1,6
Rhodobacter sp	C8RZW7	
Rhodobacter sphaeroides	A3PKH5	0
Rhodobacteraceae bacterium KLH11	B9NLC2	0,8
Rhodobacterales bacterium HTCC2255	Q0FGE0	-1,6
Roseibium sp	E2CFT5	
Roseobacter sp	A4EEU1	0,2
Sagittula stellata E-37	A3K129	-0,1
Silicibacter lacuscaerulensis ITI-1157	D0CP77	3
Silicibacter sp	Q1GJK6	-1,3
Sinorhizobium medicae	A6UC06	2,2
Sinorhizobium meliloti BL225C	E0JJX4	1,8
Thalassiobium sp	C7D981	2,6
Variovorax paradoxus	C5CPP3	2,1
Verminephrobacter eiseniae	A1WMN9	2,1
