# Supplementary material for: Insights into the evolution of sorbitol metabolism: phylogenetic analysis of SDR196C family
Source: BMC Evol Biol. 2012 Aug 16;12:147. doi: 10.1186/1471-2148-12-147 (PMC3458964; doi:10.1186/1471-2148-12-147)
Supplement: Additional file 3 — Structure of the SDH clusters among bacterial groups. SDH gene was placed in the middle to facilitate cluster visualization. Variants of the cluster among taxonomic groups are represented by numbers (1–21). [file 1471-2148-12-147-S3.pdf]

Additional File 3. Sola-Carvajal *et al*. 2011

Species	Uniprot ID	Taxonomy	Ecology	Niche/Disease	SDH linage	Cluster variant												
Rhodobacter sphaeroides	A3PKH5	α-prot Rhod	F	Photosyntetic Bacteria	6,3	1	DeoR ▶	ABC ▶	ABC ▶	ABC ▶	ABC ▶	SDH ▶	MDH ▶	tRNA ▶	TRAP ▶	TRAP ◀		
Rhodobacter capsulatus	O68112	α-prot Rhod	F	Photosyntetic Bacteria	6,1	1	DeoR ▶	ABC ▶	ABC ▶	ABC ▶	ABC ▶	SDH ▶	MDH ▶	FeoA ▶	Feo B ▶	ureidoglycolate hydrolase ◀		
Jannaschia sp	Q28N89	α-prot Rhod	F	Sea water	6,1	2	lacI ▶	ABC ▶	ABC ▶	ABC ▶	ABC ▶	SDH ▶	MDH ▶	Sialicylate hydroxilase ▶	Isomerase ▶	Cupin 2 ▶		
Paracoccus denitrificans	A1BBK7	α-prot Rhod	F	V/Extremophile	6,1	3		H Prot ▶	H Prot ▶	HAD ◀	ADH ◀	SDH ◀	ABC ◀	ABC ◀	ABC ◀	ABC ◀	lacI ◀	
Silicibacter sp	Q1GJK6	α-prot Rhod	F	Sea water	6,1	3		FAA hydrolase ▶	H Prot ▶	H Prot ▶	MDH ◀	SDH ◀	ABC ◀	ABC ◀	ABC ◀	ABC ◀	lacI ◀	
Rhizobium loti	Q98D05	α-prot Rhiz	F	S/Nitrogen fixing	6,3	4			RiboKin ◀	HAD ◀	MDH ◀	SDH ◀	ABC ◀	ABC ◀	ABC ◀	lysR ◀		
Agrobacterium radiobacter	B9JPZ8	α-prot Rhiz	F/PP	S/Tumors	6,3	5		lysR ▶	ABC ▶	ABC ▶	ABC ▶	SDH ▶	AraC ▶	MACST ◀	α/β hydrolase ◀	amidohydrolase ◀		
Agrobacterium tumefaciens	A9CES4	α-prot Rhiz	F/PP	S/Tumors	5	6		Monooxygenase ◀	homoserine DH ◀	lacI ◀	Zn DH ◀	SDH ◀	ABC ◀	FrucK ▶	Tag 6P Kinase ▶	ABC ▶		
Rhizobium etli	Q2K1R3	α-prot Rhiz	F	S/Simbiotic	5	6		sikimate DH ▶	dioxygenase ▶	lacI ◀	Zn DH ◀	SDH ◀	ABC ◀	FrucK ▶	Tag 6P Kinase ▶	SDR ◀		
Agrobacterium vitis	B9JRG2	α-prot Rhiz	F/PP	S/Tumors	6,3	7	H Prot ◀	ABC ▶	ABC ▶	ABC ▶	ABC ▶	SDH ▶	MDH ▶	H Prot ▶	RiboK ▶	tRNA ▶		
Rhizobium sp	C3MGF5	α-prot Rhiz	F	S/Nitrogen fixing	6,3	8		Response reg ▶	RiboK ◀	H Prot ◀	MDH ◀	SDH ◀	ABC ◀	ABC ◀	ABC ◀	ABC ◀	DeoR ◀	
Sinorhizobium medicae	A6UC06	α-prot Rhiz	F	S/Nitrogen fixing	6,3	8		lysR ▶	Hexokinase ◀	HAD ◀	MDH ◀	SDH ◀	ABC ◀	ABC ◀	ABC ◀	ABC ◀	DeoR ◀	
Mesorhizobium sp	Q11BS6	α-prot Phyll	F	S/Nitrogen fixing	6,3	9		lysR ▶	ABC ▶	ABC ▶	ABC ▶	SDH ▶	SDR ▶	HAD ▶	Hexokinase ▶	rhodanese like protein ▶		
Azospirillum sp	D3P2X3	α-prot Rhodosp	F	S/Nitrogen fixing	6,3	10		NADH DH ▶	NADH DH ▶	FrucK ◀	MDH ◀	SDH ◀	ABC ◀	ABC ◀	ABC ◀	ABC ◀	NagC ▶	
Acidiphilium cryptum	A5FVQ6	α-prot Acet	F	Iron contaminated habitats	4	11	lysR ▶	ABC ▶	ABC ▶	ABC ▶	SDR ▶	SDH ▶	Glu DH ▶	Pgluc DH ▶	2DGPH Aldo ▶	MFS ▶		
Ochrobactrum anthropi	A6X5Q1	α-prot Brucell	P	Septicaemia	5	12		Proline DH ◀	AsnC ▶	MDH ◀	ADH ◀	SDH ◀	ABC ◀	ABC ◀	ABC ◀	lysR ◀		
Variovorax paradoxus	C5CPP3	β-prot Comamon	F	S	2	13	lysR ▶	ABC ▶	ABC ▶	ABC ▶	MDH ▶	SDH ▶	Aldolase ▶	FrucK ▶	AraC ▶	Xkinase ▶		
Verminephrobacter eiseniae	A1WMN9	β-prot Burkh	C	Earthworms nephridia	2	14		Asuccinate synt ◀	tRNA synthase ◀	Xkinase ◀	AraC ◀	SDH ◀	MDH ◀	ABC ◀	ABC ◀	ABC ◀	lysR ◀	
Burkholderia phytofirmans	B2SYA3	β-prot Burkh	F	S	1	15		HAD ◀	ABC ◀	ABC ◀	ABC ◀	SDH ◀	Ferric uptake ▶	ABC ▶	ABC ▶	ABC ▶		
Burkholderia xenovorans	Q13UM4	β-prot Burkh	F	S/Nitrogen fixing	1	15		HAD ◀	ABC ◀	ABC ◀	ABC ◀	SDH ◀	Ferric uptake ▶	ABC ▶	ABC ▶	ABC ▶		
Burkholderia phymatum	B2JDG4	β-prot Burkh	F	Nitrogen fixing	1	15		ABC ◀	ABC ◀	ABC ◀	Ferric Uptake ◀	SDH ▶	ABC ▶	ABC ▶	ABC ▶	HAD ▶		
Burkholderia ambifaria	B1YVT1	β-prot Burkh	F/P	V/Cystic Fibrosis	1	16	ABC ◀	ABC ◀	lysR ◀	Tag biP Aldo ◀	RiboK ◀	SDH ◀	Ferric Uptake ▶	ABC ▶	ABC ▶	ABC ▶		
Burkholderia cenocepacia	Q1BU16	β-prot Burkh	F/P	V/Cystic Fibrosis	1	16	ABC ◀	ABC ◀	lysR ◀	Tag biP Aldo ◀	RiboK ◀	SDH ◀	Ferric Uptake ▶	ABC ▶	ABC ▶	ABC ▶		
Burkholderia sp	Q39DE3	β-prot Burkh	F	Organic compounds	1	16	ABC ◀	ABC ◀	lysR ◀	Tag biP Aldo ◀	RiboK ◀	SDH ◀	Ferric Uptake ▶	ABC ▶	ABC ▶	ABC ▶		
Burkholderia vietnamiensis	A4JHF0	β-prot Burkh	F	Paddies	1	16	ABC ◀	ABC ◀	lysR ◀	Tag biP Aldo ◀	RiboK ◀	SDH ◀	Ferric Uptake ▶	ABC ▶	ABC ▶	ABC ▶		
Burkholderia multivorans	A9AFX9	β-prot Burkh	F/P	V/Cystic Fibrosis	1	16	ABC ◀	ABC ◀	lysR ◀	Tag biP Aldo ◀	RiboK ◀	SDH ◀	Ferric Uptake ▶	ABC ▶	ABC ▶	ABC ▶		
Burkholderia mallei	A3MHB9	β-prot Burkh	P	Glanders	1	17		ABC ◀	ABC ◀	ABC ◀	Ferric Uptake ◀	SDH ▶	kdgK ▶	Pseudo ▶	ABC ▶	ABC ▶	ABC ▶	HAD ▶
Burkholderia thailandensis	Q2T0Q1	β-prot Burkh	F	S	1	17		ABC ◀	ABC ◀	ABC ◀	Ferric Uptake ◀	SDH ▶	kdgK ▶	Tag 6P Kinase ▶	ABC ▶	ABC ▶	ABC ▶	HAD ▶
Chromohalobacter salexigens	Q1QZV8	γ-prot Halom	F	Salterns	3	18	lysR ▶	ABC ▶	ABC ▶	ABC ▶	HAD ▶	SDH ▶	PfkB ▶	Tag biP Aldo ▶	SDR ▶	Fruct transp ▶	MDH ▶	
Halomonas elongata DSM 2581	E1VCL7	γ-prot Halom	F	High salinity water	3	19	DeoR ▶	ABC ▶	ABC ▶	ABC ▶	HAD ▶	SDH ▶	FrucK ▶	MDH ▶	Xkinase ▶	Aldolase ▶		
Pseudomonas syringae pv	Q88AM6	γ-prot Pseud	F/PP	Saprofit/various	6,3	20		Glut synt ◀	thiamin biosynthesis ▶	GTP BP ▶	DeoR ◀	SDH ◀	ABC ◀	AraC ▶	Na/H antiporter ◀	H Prot ◀		
Pseudomonas fluorescens	C3K8P4	γ-prot Pseud	C	Plant	6,3	21		Phosphorilase ◀	TSX channel ◀	AraC ◀	lysR ▶	SDH ▶	H Prot ◀	H Prot ▶	H Prot ▶	H Prot ▶		

Taxonomy legend		Ecology legend		Niche/Disease legend	
Burkh	Burkholderiac	P	Pathogen	V	Various
Rhiz	Rhizobiaceae	F	Free living	S	Soil
Pseud	Pseudomonac	PP	Plant Pathogen		
Rhod	Rhodobacteri	C	Comensal		
Acet	Acetobacteriaceae				
Halom	Halomonadaceae				
Phyll	Phyllobacteriaceae				
Brucell	Brucellaceae				
Ocean	Oceanospirillales				
Comamon	Comamonadaceae				
Rhodosp	Rhodospirillaceae				

ABC	ABC Transporter	Glu DH	Glucose dehydrogenase	Shikimate DH	Shikimate 5-dehydrogenase
SDH	Sorbitol dehydrogenase	Pgluc DH	Phosphogluconate dehydrogenase	Response reg	Two component response regulator
MDH	Mannitol dehydrogenase	2DGPH Aldo	2-deoxy-3-dexyphosphogluconate aldolase	Rhodanese like protein	sulfurtransferase
tRNA	Transference RNA	Ferric uptake	Ferric uptake protein	NADH DH	NADH dehydrogenase
FeoA	Ferric transport protein	lysR	lysR type transcription regulator	NagC	NagC type transcription regulator
HAD	Haloacid dehalogenase-like hydrolases	Tag biP Aldo	Tagatose biphosphate aldolase	MFS	Major Facilitator Superfamily transporter
ADH	Alcohol dehydrogenase	Xkinase	Xylulokinase	Proline DH	Proline Dehydrogenase
RiboKin	Ribitol/Ribokinase	PfkB	2-keto-3-deoxygluconate kinase	AsnC	leucine responsive transcription regulator
SDR	Short chain dehydrogenase	GTP BP	GTP binding protein	Asuccinate syn	Adenylsuccinate synthase
AraC	Arabinose related transcription regulator	DeoR	Sugar related transcription regulator	Fruc Transp	Fructose transporter
MACST	Metil accepting Chemotaxis Sensory transducer	TRAP	Dicarboxilate transporter	Glut synt	Glutamine synthetase
lacI	Lactose related transcription regulator	Feo B	Ferrous transport protein	thiamin biosynthesis	Thiamin biosynthesis related protein
Zn DH	Zn dependent dehydrogenase	Isomerase	5-carboxymethyl-2-hydroxymuconate delta-isomerase	Na/H antiporter	Sodium/hydrogen exanger
FrucK	Fructokinase	FAA hydrolase	Fumarylacetoacetate hydrolase	TSX channel	Nucleotide specific channel forming protein
Tag 6P Kinase	Tagatose 6 phosphate kinase	Homoserine DH	Homoserine dehydrogenase	CH kinase	carbohydrate kinase
H Prot	Hypotetical protein	Monoxygenase	Antibiotic biosynthesis monoxygenase	kdgK	2-keto-3-deoxygluconate kinase
Hexokinase	Hexokinase	Dioxygenase	4-hydroxyphenylpyruvate dioxygenase		

Additional File 3. Sola-Carvajal *et al*. 2011
