# Supplementary figures and images for: Insights into the evolution of sorbitol metabolism: phylogenetic analysis of SDR196C family
Source: BMC Evol Biol. 2012 Aug 16;12:147. doi: 10.1186/1471-2148-12-147 (PMC3458964; doi:10.1186/1471-2148-12-147)

# SDH Tree Bayesian

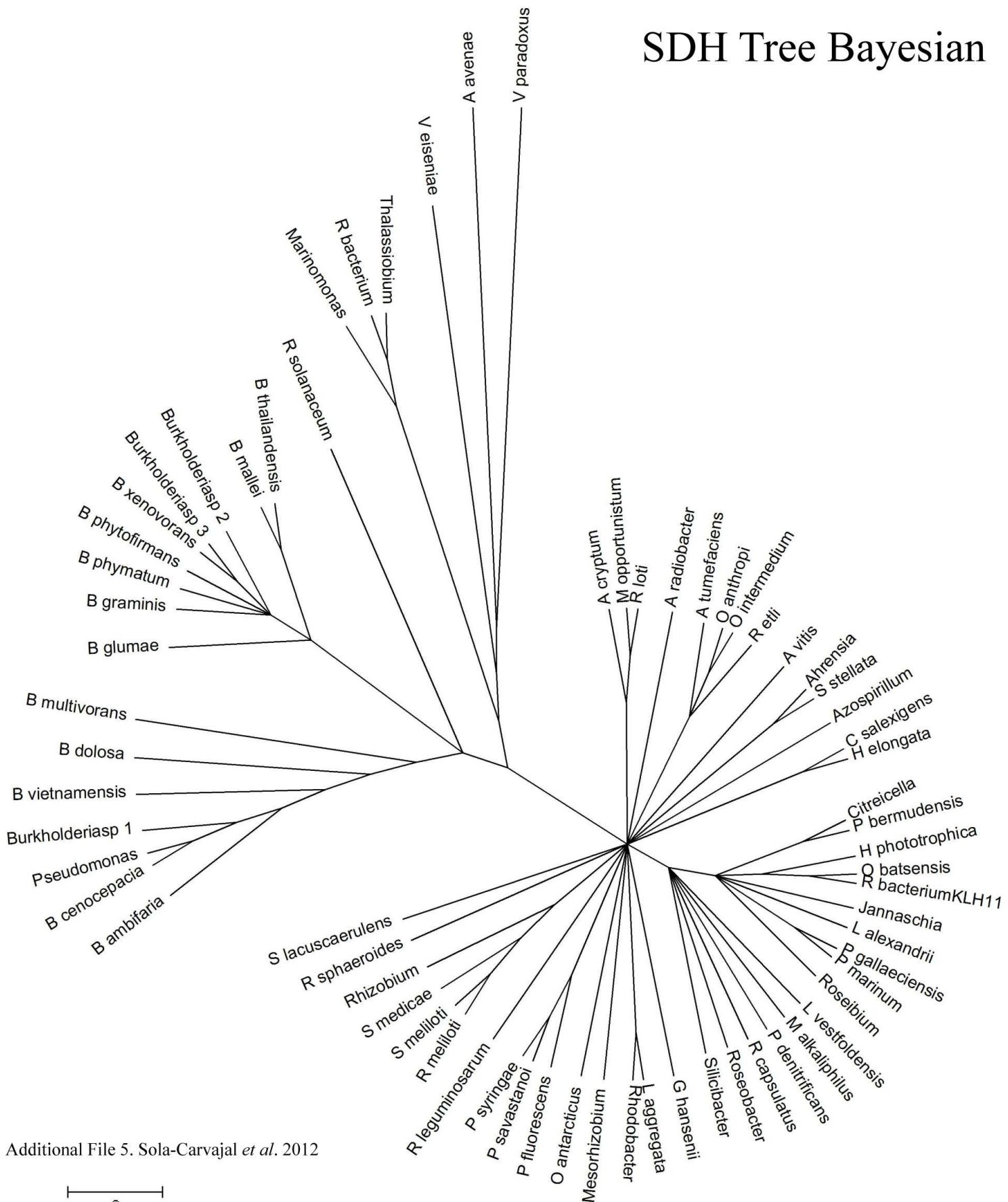

# Maximum likelihood

Additional File 5. Sola-Carvajal *et al.* 2012.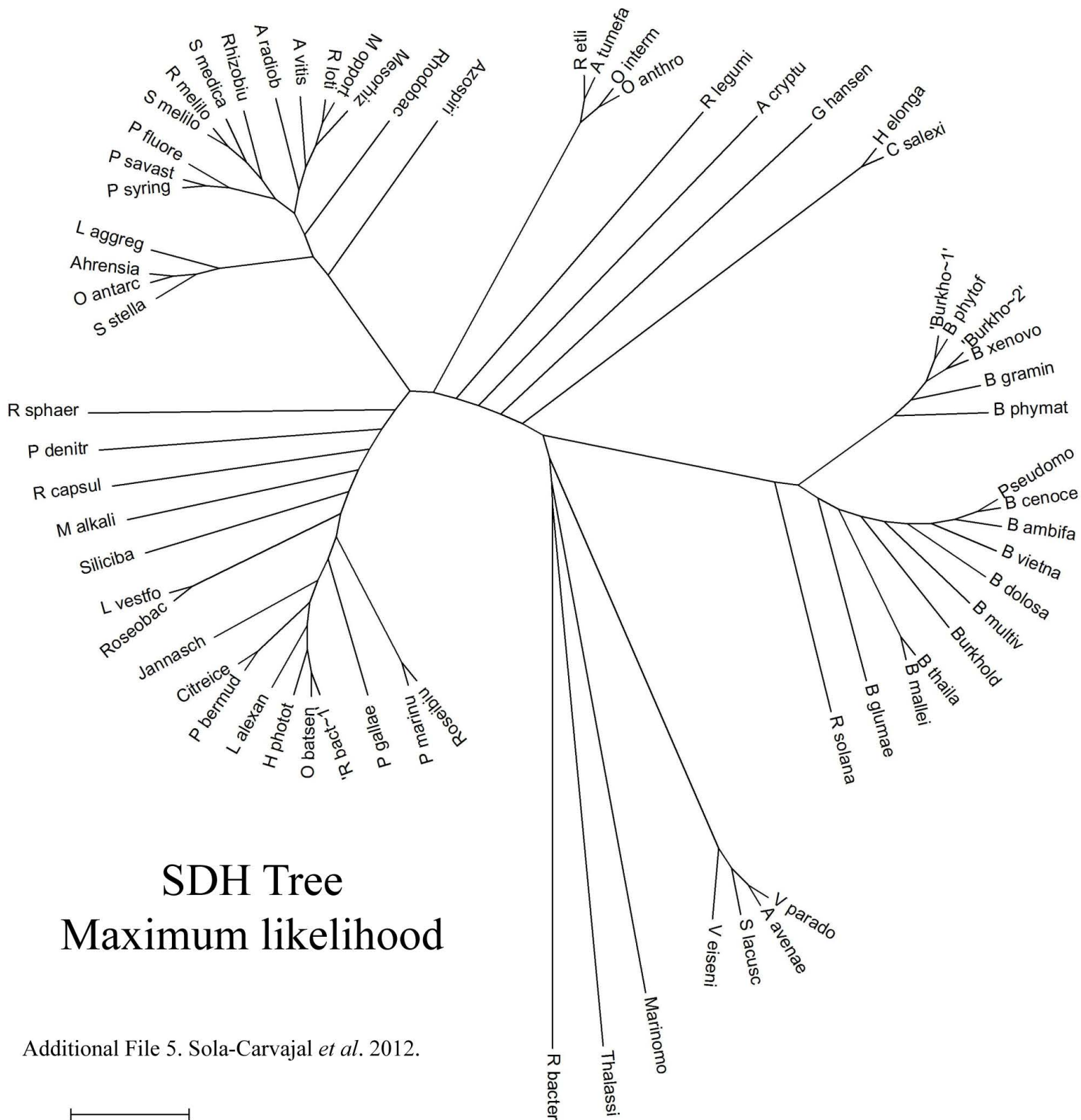

Supplement: Additional file 5 — Phylogenetic trees of bacteria containing SDH gene. The file shows two phylogenetic trees of SDH gene using Bayesian and Maximun Likelihood, as tree building methods. [file 1471-2148-12-147-S5.pdf]

# 16S Tree Bayesian

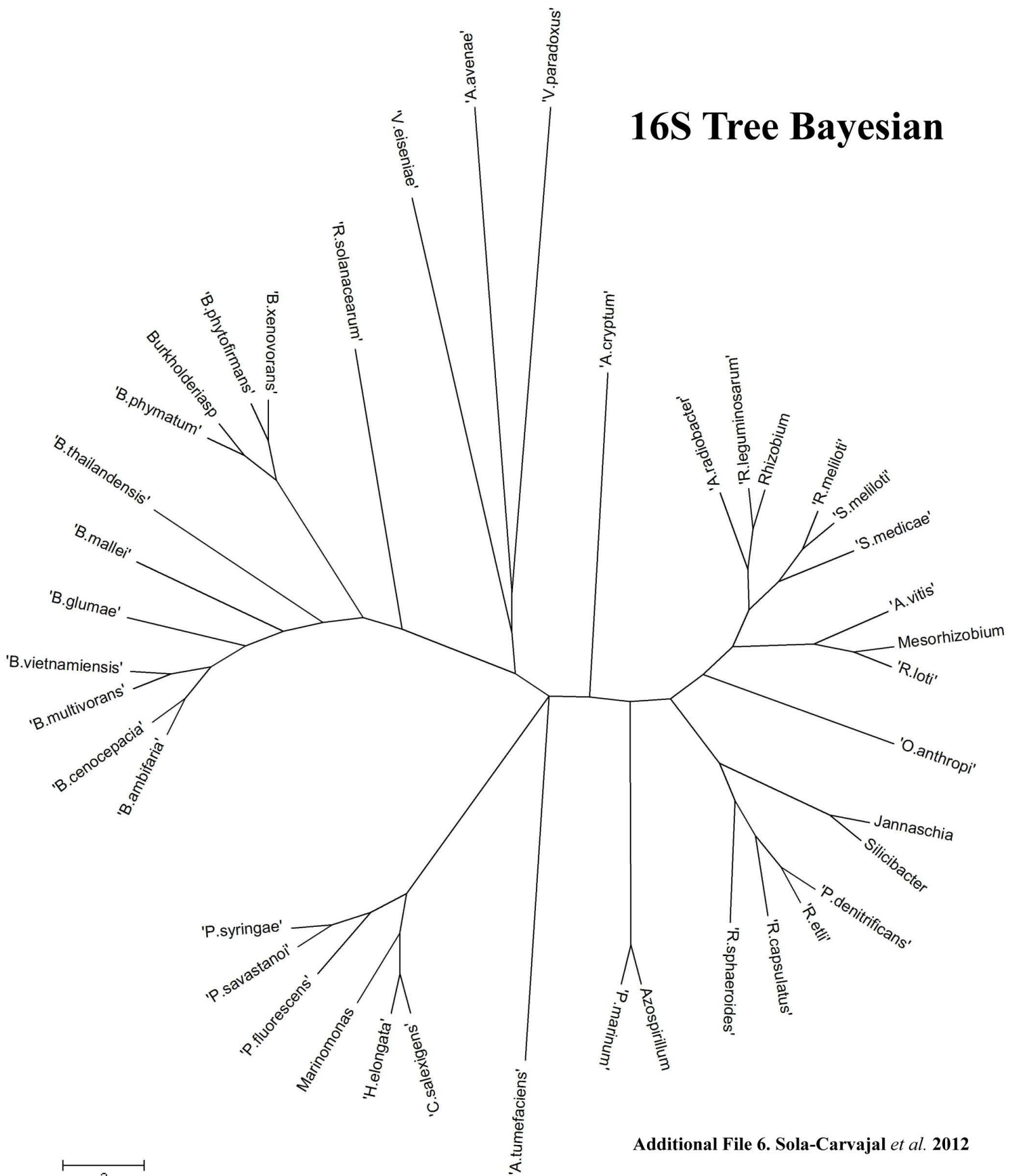

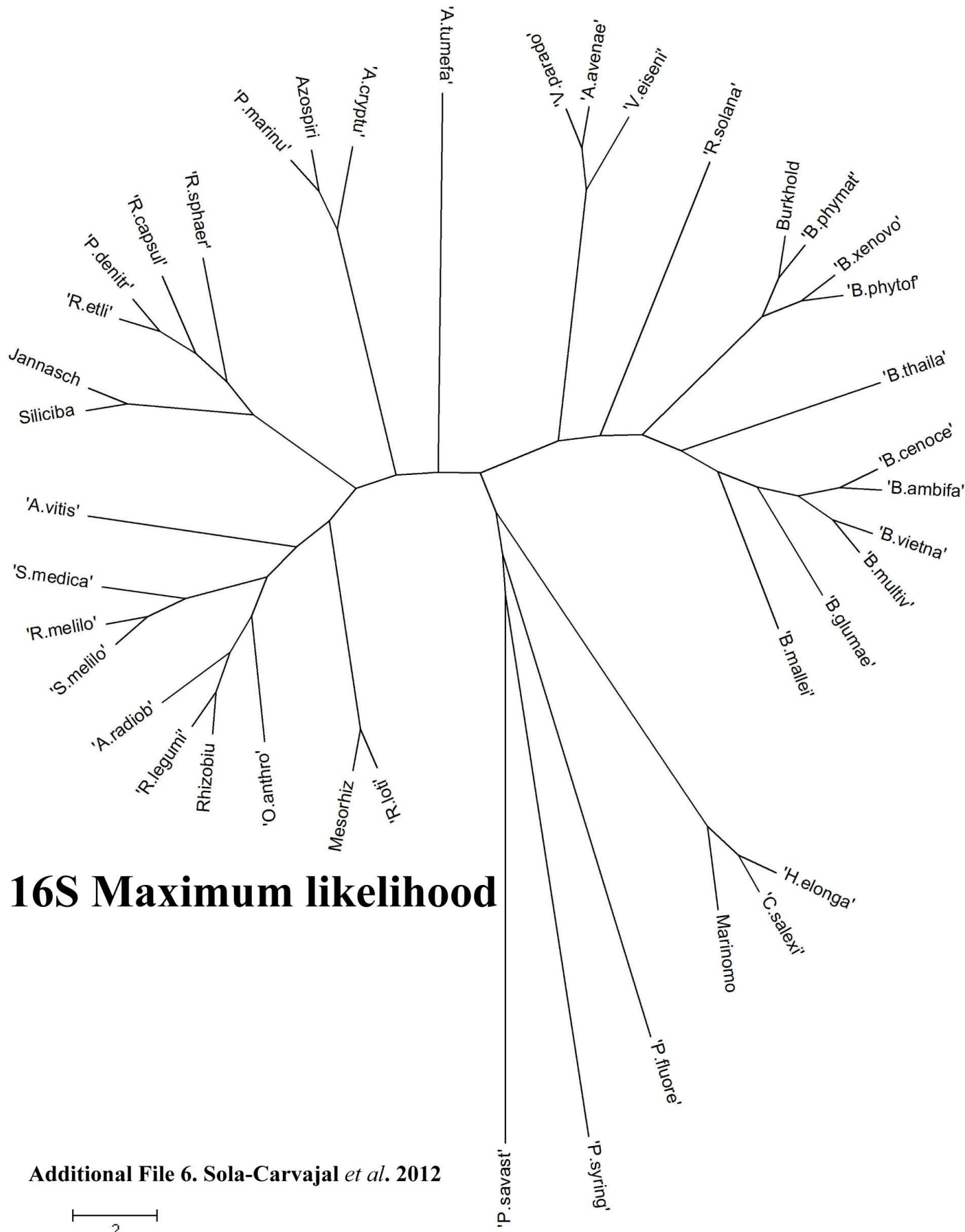

Supplement: Additional file 6 — Phylogenetic trees (16S rRNA) of bacteria containing the SDH gene. The file contained two phylogenetic trees of 16S rRNA of bacteria containing SDH gene, using Bayesian and Maximum Likelihood, as tree building methods. [file 1471-2148-12-147-S6.pdf]

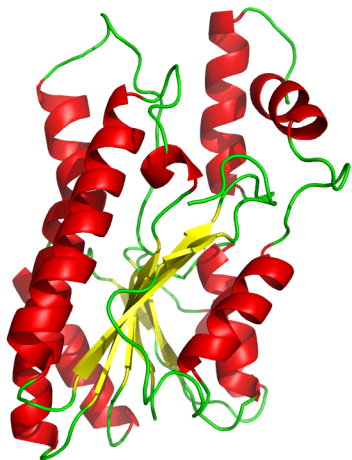

Lineage 1

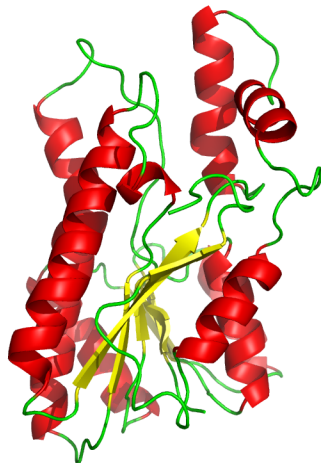

Lineage 2

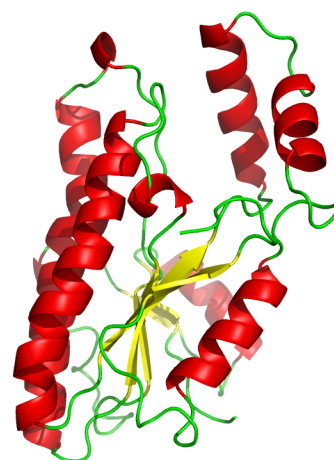

Lineage 3

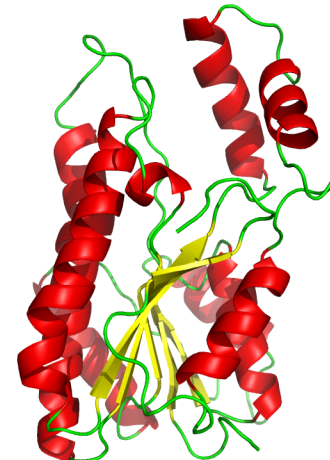

Lineage 4

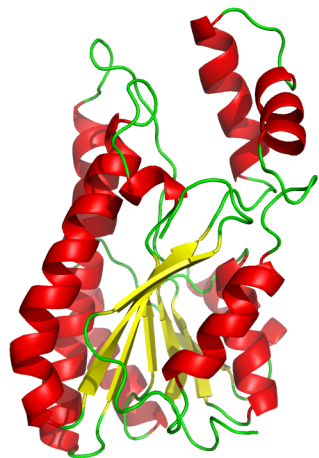

Lineage 5

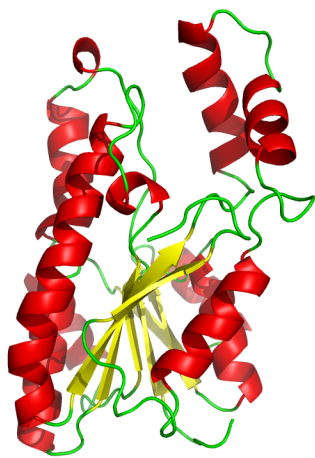

Lineage 6.1

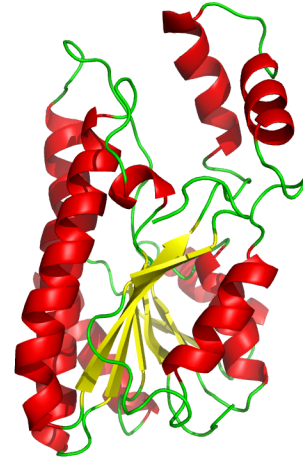

Lineage 6.2

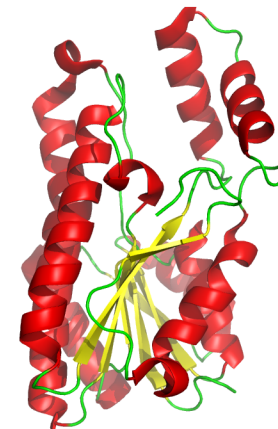

Lineage 6.3

Supplement: Additional file 7 — Molecular modeling of SDH enzymes from the six lineages described in bacterial SDH. The proteins were modeled with Geno3D [49] and rendered by PyMol [50]. Selected proteins were Uniprot codes: lineage 1, A3MHB9; lineage 2, A1WMN; lineage 3 E1VCL7; lineage 4, A5FVQ; lineage 5, A9CES4; lineage 6.1, O68112; lineage 6.2, A3K129; lineage 6.3, A3PKH5. [file 1471-2148-12-147-S7.pdf]

## Lineage 1

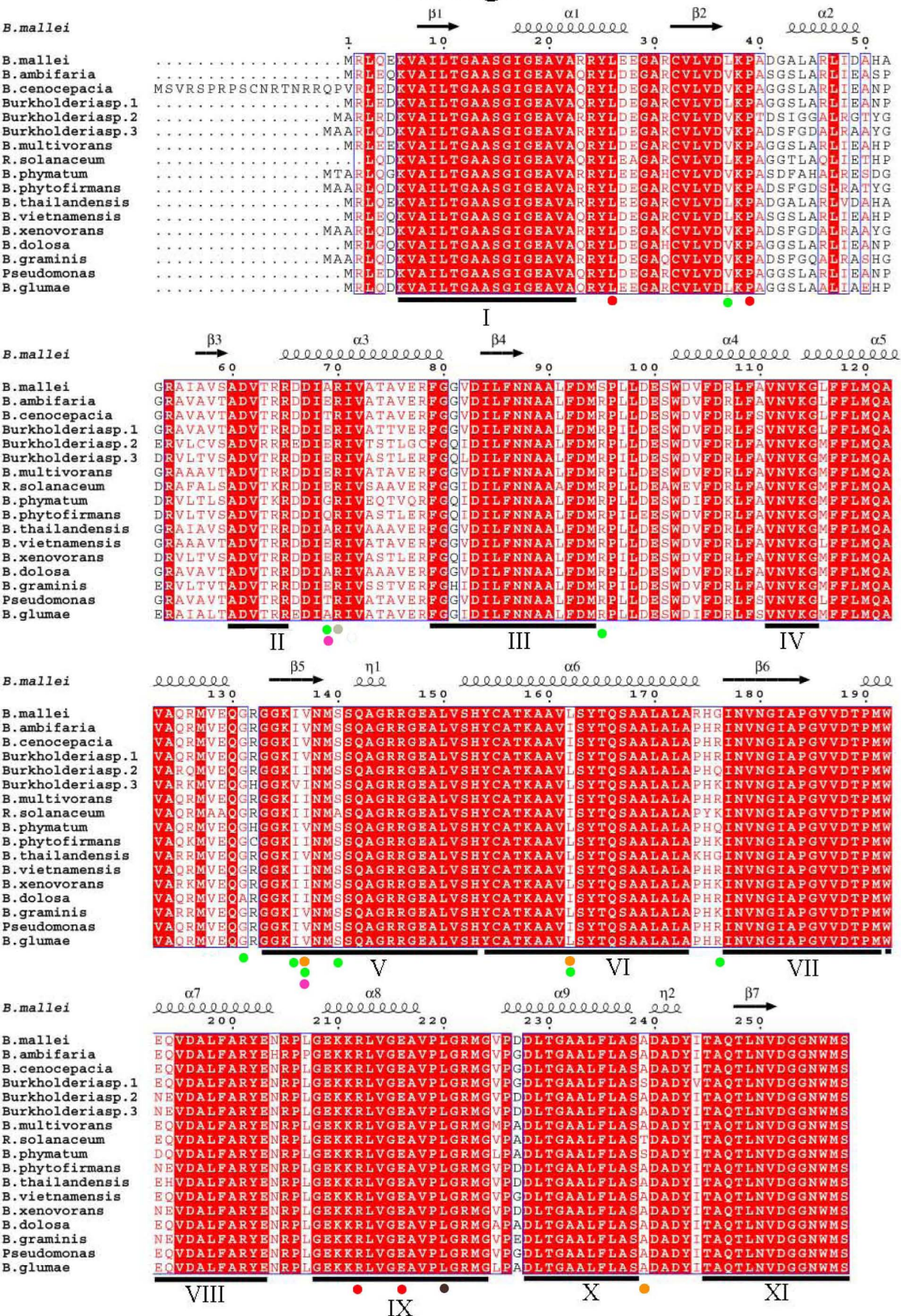

# Lineage 2

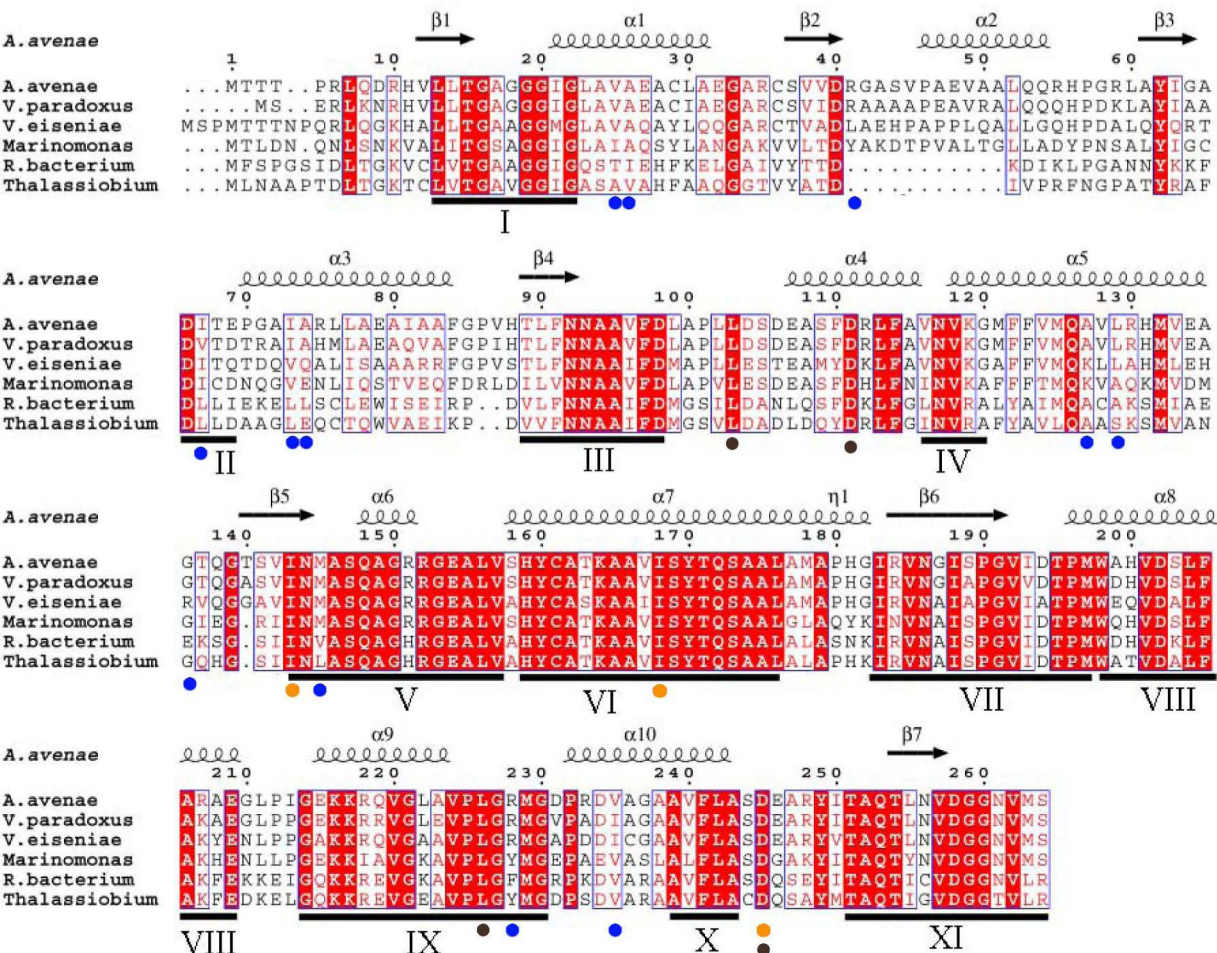

# Lineage 3

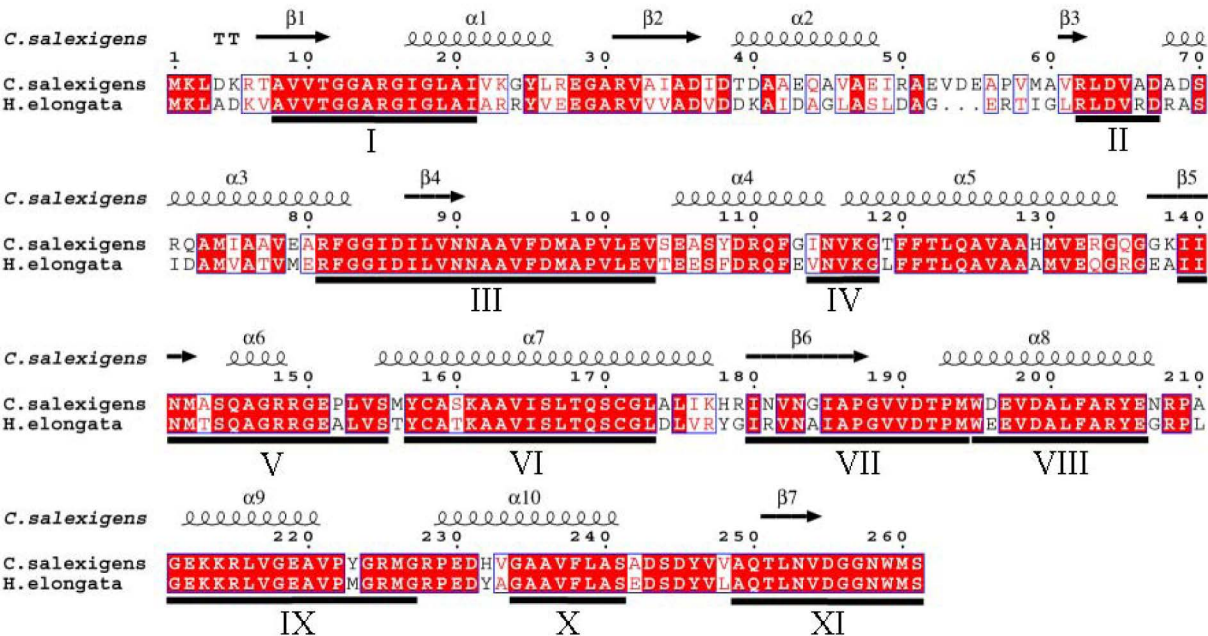

# Lineage 4

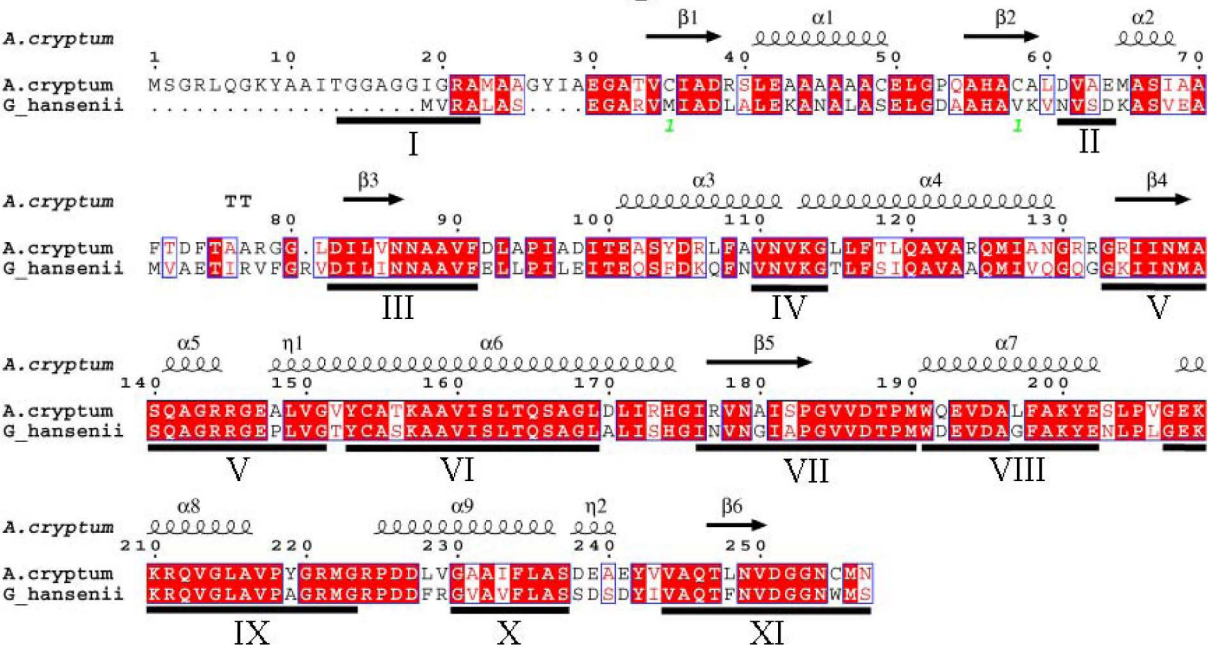

# Lineage 5

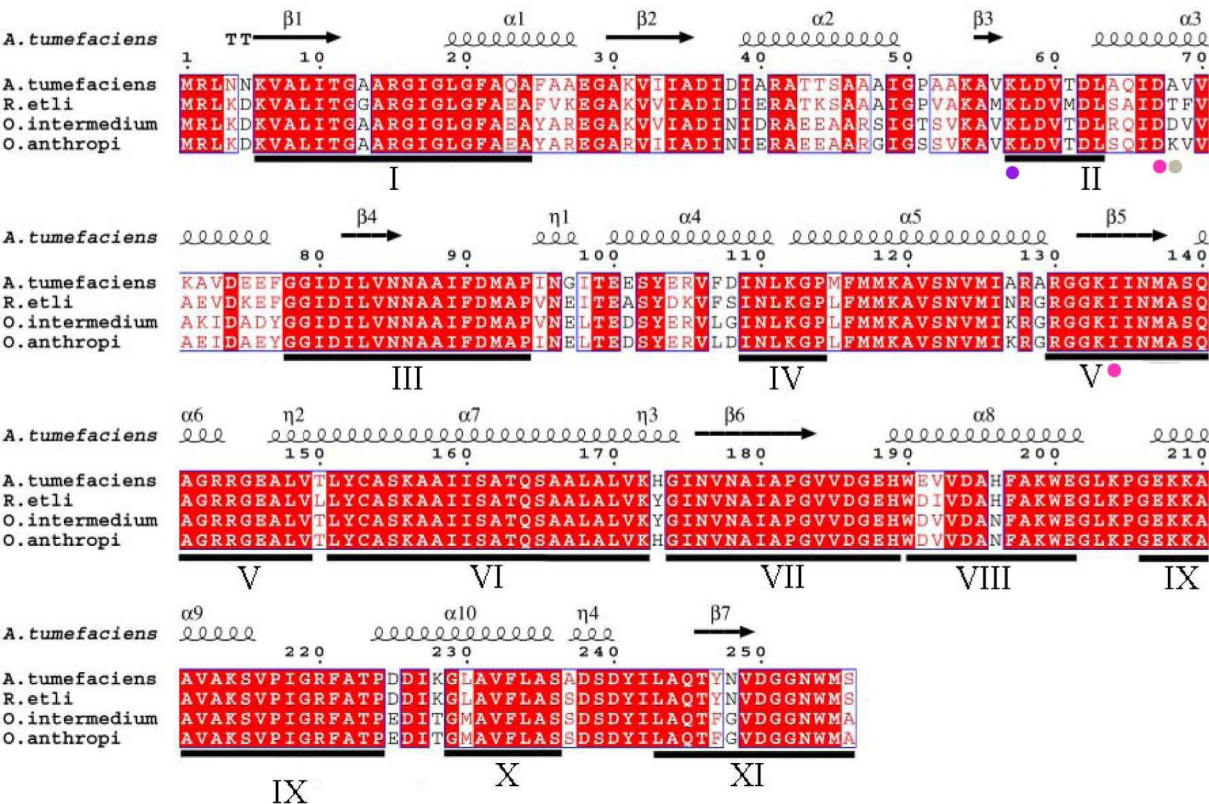

# Lineage 6

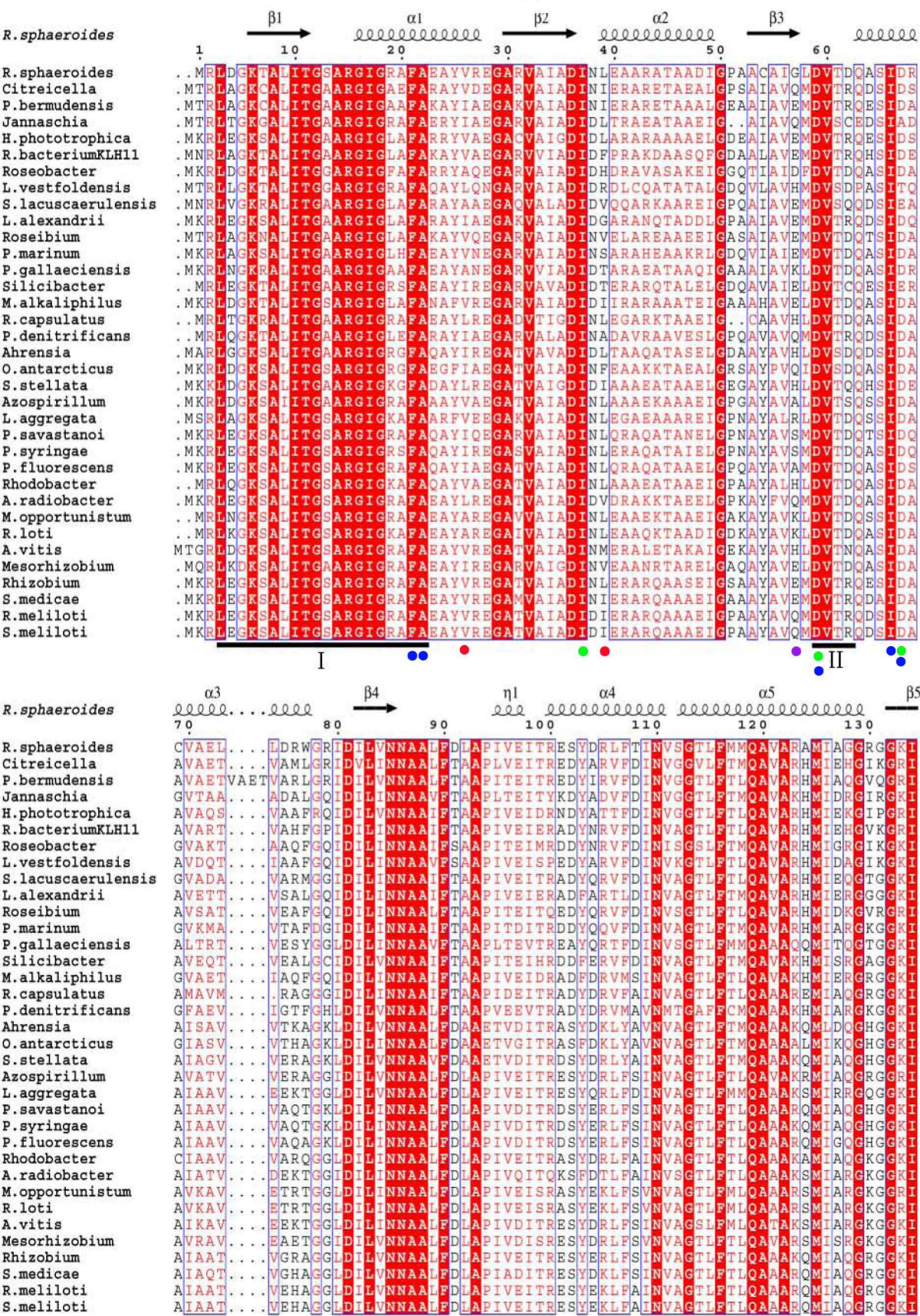

# Lineage 6 (cont).

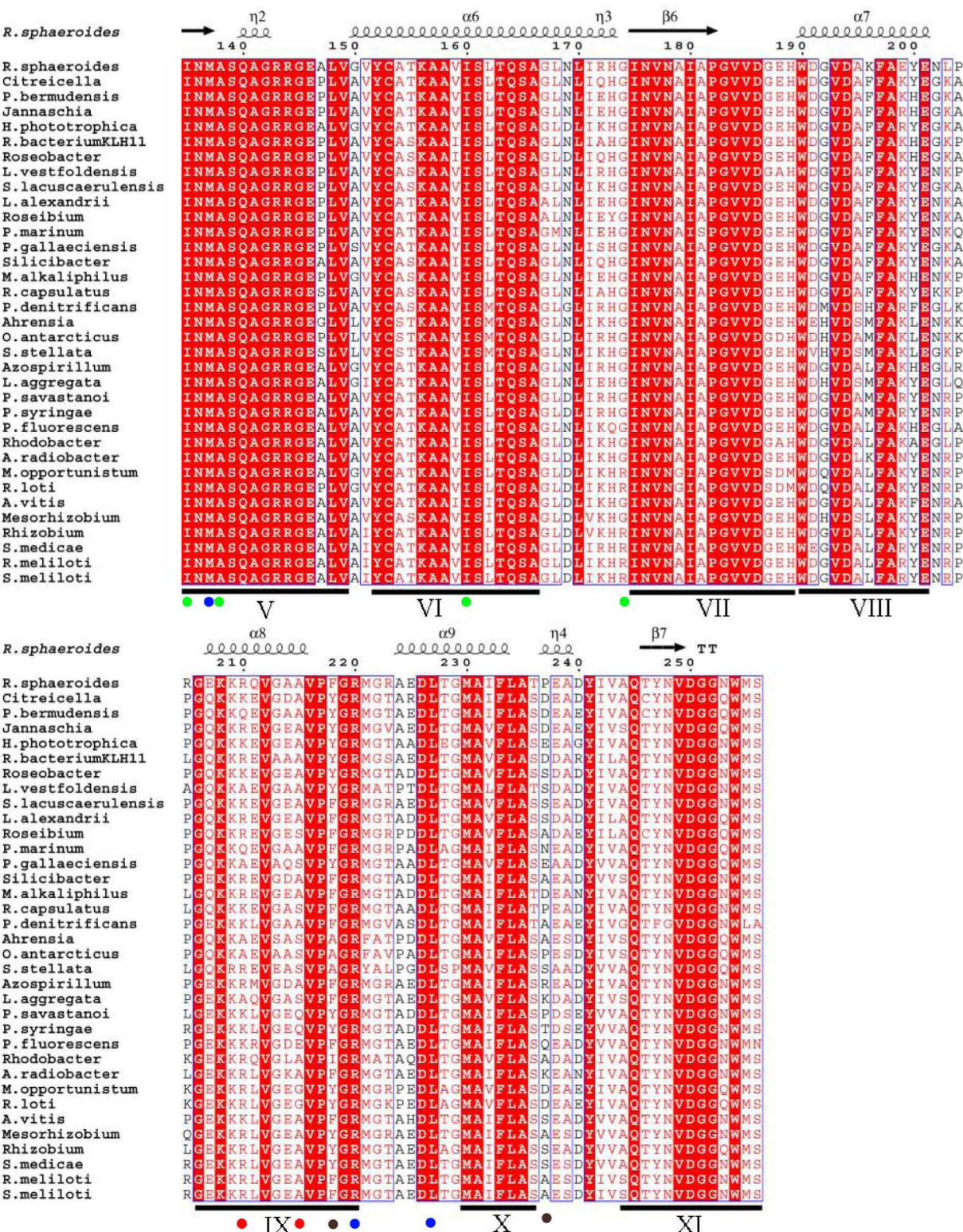

# Lineage 6.1

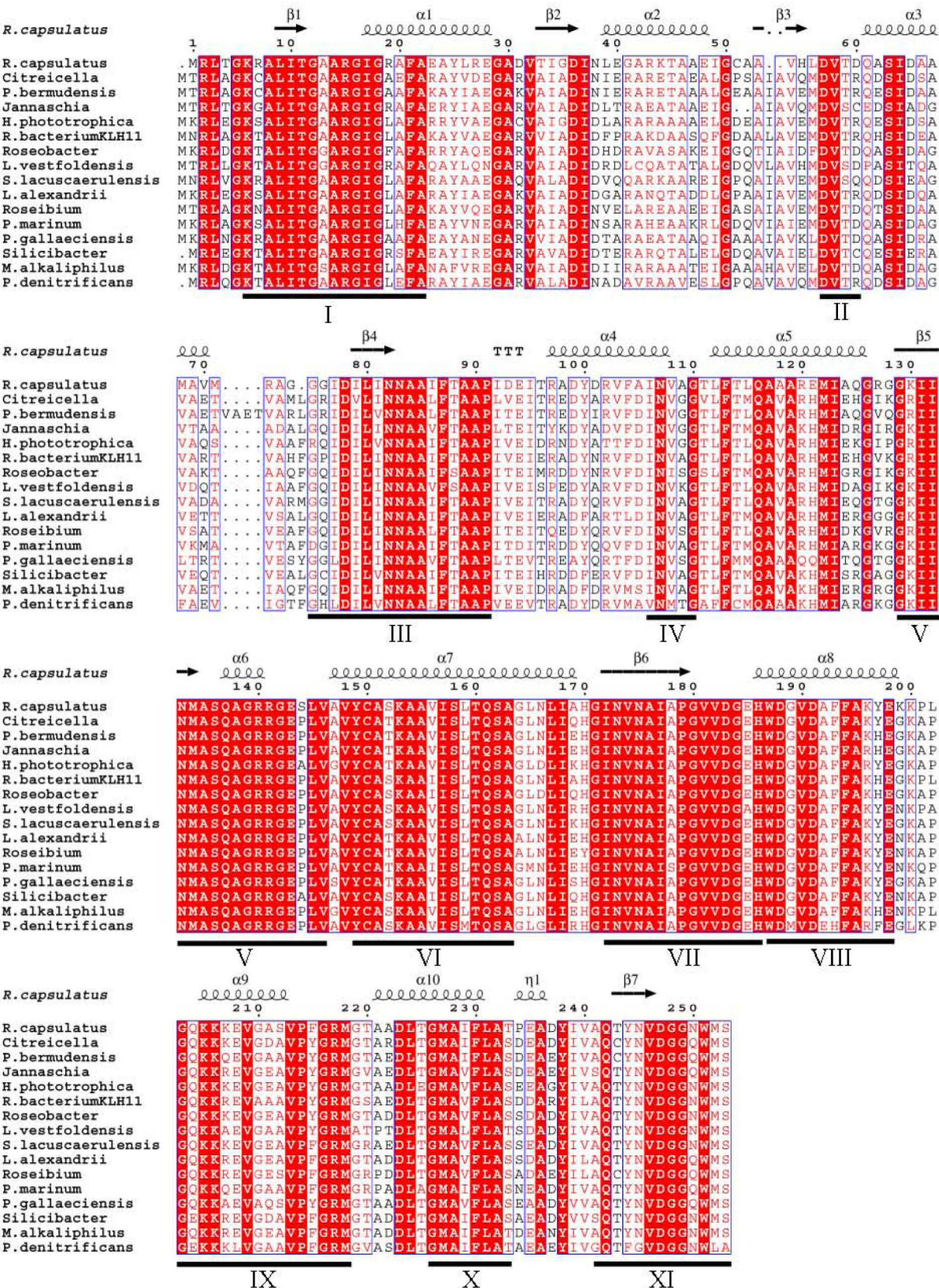

# Lineage 6.2

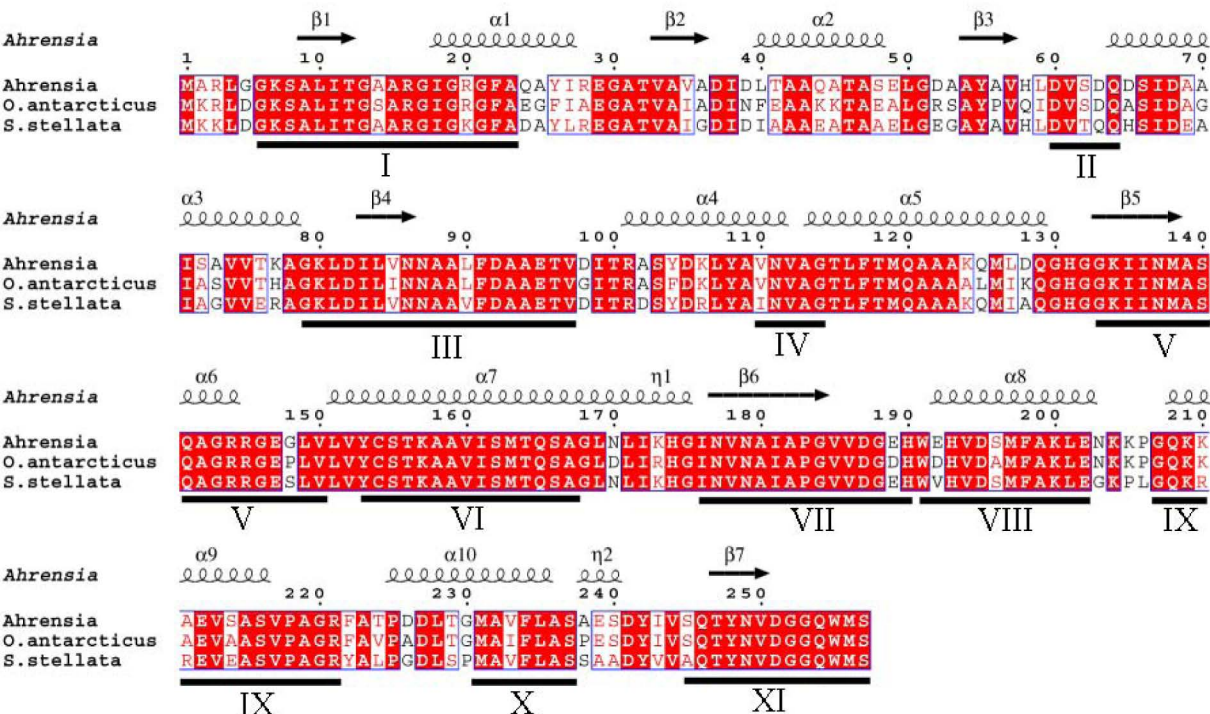

# Lineage 6.3

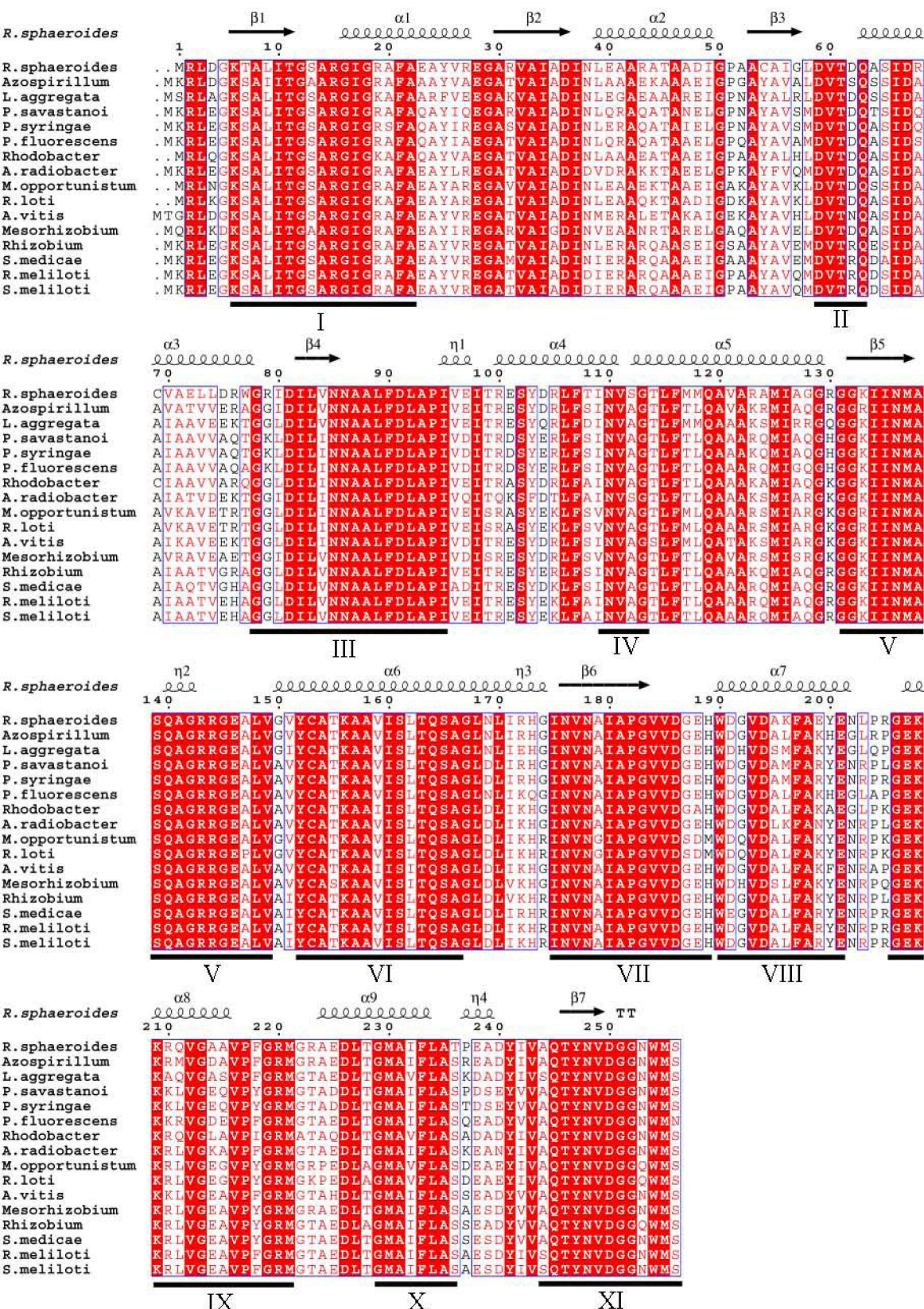

Supplement: Additional file 8 — Multiple sequence alignment of bacterial SDH proteins. ESPript [37] output was obtained with the aligned sequences from Additional file 1. Strictly conserved residues have a solid background. Symbols above sequences represent the secondary structure. Conserved structural blocks (I-XI) are shown below the sequences. Circles represent the divergent amino acids according to the following color code: Grey; conserved amino acids in Lineage I and divergent in Lineage V, Red; conserved amino acids in Lineage I and divergent in Lineage VI, Orange; conserved amino acids in Lineage II and divergent in Lineage I, Black; conserved amino acids in Lineage II and divergent in Lineage VI, Pink; conserved amino acids in Lineage V and divergent in Lineage I, Purple; conserved amino acids in Lineage V and divergent in Lineage VI, Green; conserved amino acids in Lineage VI and divergent in Lineage I, Blue; conserved amino acids in Lineage VI and divergent in Lineage II. [file 1471-2148-12-147-S8.pdf]
