# Supplementary material for: Insights into the evolution of sorbitol metabolism: phylogenetic analysis of SDR196C family
Source: BMC Evol Biol. 2012 Aug 16;12:147. doi: 10.1186/1471-2148-12-147 (PMC3458964; doi:10.1186/1471-2148-12-147)
Supplement: Additional file 9 — Predicted critical amino acid sites responsible for functional divergence. The table represents the values of Qk of all pair analyses. Values with a Qk > 0.75 are indicated with a red background. [file 1471-2148-12-147-S9.pdf]

Lineages	Position (k)	Q_k
1_2	**168**	**0,971332**
	**270**	**0,838171**
	**193**	**0,814521**
	207	0,627376
	91	0,542016
	156	0,468716
	57	0,444574
	86	0,428651
	171	0,353074
	51	0,352479
	39	0,346183
	52	0,346183
	159	0,346183
	149	0,343239
	81	0,339697
	89	0,339697
	233	0,336537
	244	0,334842
	273	0,334479
	88	0,329781
	247	0,329781
	44	0,327504
	93	0,320486
	90	0,319359
	56	0,317184
	53	0,316126
	105	0,269636
	237	0,269636
	262	0,269636
	271	0,269636
	280	0,269636
	120	0,268354
	183	0,268354
	240	0,268354
	166	0,267268
	40	0,265866
	170	0,265866
	281	0,265866
	145	0,26485
	42	0,264343
	109	0,264343
	131	0,26371
	108	0,262257
	43	0,261879
	148	0,261879
	83	0,261124
	92	0,261124
	137	0,260119
	150	0,260119
	253	0,260119
	214	0,259179
	260	0,259179
	95	0,237218
	122	0,213794
	126	0,213794
	128	0,213794
	140	0,213794
	143	0,213794
	144	0,213794
	176	0,213794
	236	0,213794
	272	0,213794

Lineages	Position (k)	Q_k
1_5	**91**	**0,879067**
	**90**	**0,843482**
	**168**	**0,764001**
	51	0,722129
	207	0,683627
	256	0,678846
	193	0,586687
	158	0,570593
	151	0,554066
	55	0,550179
	44	0,491257
	88	0,491257
	92	0,491257
	132	0,491257
	183	0,491257
	43	0,490603
	56	0,490603
	126	0,490603
	135	0,490603
	229	0,490603
	261	0,490603
	263	0,490603
	280	0,490603
	281	0,490603
	289	0,490603
	85	0,489207
	143	0,488121
	130	0,477144
	152	0,466214
	80	0,461644
	205	0,460982
	142	0,46054
	46	0,379634
	116	0,379634
	127	0,379634
	154	0,379634
	171	0,379634
	275	0,373724
	121	0,373006
	235	0,372791
	238	0,372791
	167	0,372647
	39	0,294932
	40	0,294932
	42	0,294932
	47	0,294932
	48	0,294932
	50	0,294932
	52	0,294932
	53	0,294932
	54	0,294932
	57	0,294932
	81	0,294932
	82	0,294932
	83	0,294932
	86	0,294932
	89	0,294932
	93	0,294932
	105	0,294932
	108	0,294932
	109	0,294932
	110	0,294932

Lineages	Position (k)	Q_k
1_6	**168**	**0,99997**
	**193**	**0,997301**
	**90**	**0,99656**
	**55**	**0,993947**
	**207**	**0,983325**
	**57**	**0,860848**
	**44**	**0,806583**
	**171**	**0,790701**
	**243**	**0,772787**
	**157**	**0,766211**
	**121**	**0,764108**
	**167**	**0,762598**
	**247**	**0,756761**
	156	0,726211
	91	0,70964
	132	0,683462
	130	0,645804
	233	0,641185
	237	0,63947
	111	0,637596
	140	0,616649
	273	0,605843
	251	0,593769
	92	0,580373
	124	0,575411
	265	0,570778
	56	0,564409
	202	0,542914
	229	0,541674
	248	0,539015
	146	0,529781
	93	0,509757
	81	0,509213
	183	0,50885
	166	0,508124
	232	0,50758
	180	0,505584
	271	0,505584
	286	0,503225
	159	0,502862
	79	0,498107
	154	0,484594
	145	0,483597
	46	0,48321
	277	0,480832
	192	0,480095
	131	0,478989
	188	0,477516
	149	0,475122
	42	0,451343
	257	0,450786
	261	0,450228
	134	0,449857
	246	0,449671
	244	0,447814
	52	0,423112
	135	0,423112
	128	0,422461
	269	0,422368
	137	0,422275
	53	0,421995
	236	0,420415

Lineages	Position (k)	Q_k
2_5	132	0,002269
	135	0,002262
	229	0,002262
	270	0,002236
	55	0,001824
	156	0,001824
	158	0,001824
	57	0,001731
	86	0,001671
	80	0,001669
	256	0,001653
	151	0,001406
	39	0,001385
	52	0,001385
	159	0,001385
	149	0,001376
	81	0,001364
	89	0,001364
	233	0,001354
	244	0,001348
	273	0,001347
	142	0,001332
	247	0,001332
	46	0,001323
	238	0,001318
	90	0,001315
	85	0,001304
	93	0,001302
	235	0,001302
	53	0,001288
	105	0,001141
	152	0,001141
	237	0,001141
	262	0,001141
	271	0,001141
	116	0,001137
	120	0,001137
	240	0,001137
	166	0,001133
	40	0,001129
	170	0,001129
	145	0,001126
	205	0,001126
	42	0,001124
	109	0,001124
	127	0,001122
	130	0,001122
	131	0,001122
	154	0,001122
	275	0,001122
	108	0,001118
	148	0,001117
	83	0,001114
	137	0,001111
	150	0,001111
	253	0,001111
	214	0,001108
	260	0,001108
	261	0,001067
	263	0,001062
	126	0,001058
	289	0,001058

Lineages	Position (k)	Q_k
2_6	**270**	**0,999504**
	**55**	**0,998208**
	**39**	**0,971148**
	**89**	**0,966373**
	**132**	**0,919387**
	**86**	**0,842282**
	**40**	**0,806926**
	**170**	**0,806926**
	**251**	**0,805435**
	**157**	**0,797269**
	**108**	**0,790534**
	**148**	**0,788751**
	**83**	**0,78515**
	**150**	**0,78028**
	**253**	**0,78028**
	**260**	**0,775643**
	**124**	**0,772808**
	**88**	**0,755648**
	46	0,742414
	243	0,704825
	229	0,704472
	180	0,620745
	277	0,55859
	257	0,480435
	134	0,478002
	246	0,477515
	262	0,476616
	120	0,474073
	280	0,473738
	121	0,464213
	144	0,464213
	176	0,464213
	259	0,461618
	263	0,461618
	281	0,455107
	48	0,452292
	110	0,452292
	109	0,452225
	154	0,449099
	50	0,448157
	141	0,448157
	167	0,448157
	203	0,448157
	218	0,439396
	214	0,425177
	51	0,413622
	135	0,408408
	111	0,399556
	140	0,359867
	195	0,337735
	205	0,337544
	255	0,337284
	129	0,336173
	105	0,299287
	265	0,292983
	206	0,288097
	130	0,284907
	139	0,279505
	254	0,277655
	184	0,276835
	279	0,276426
	204	0,276221

Cluster	Position (k)	Q_k
5_6	80	0,7524934
	263	0,710589
	157	0,704485
	206	0,679776
	51	0,506535
	57	0,479502
	205	0,406672
	243	0,393896
	247	0,381558
	158	0,379401
	88	0,376748
	280	0,371287
	281	0,371287
	143	0,366605
	156	0,359858
	256	0,346779
	238	0,334224
	85	0,323904
	151	0,31917
	233	0,308668
	237	0,307743
	111	0,306734
	140	0,295736
	127	0,292149
	273	0,290241
	251	0,284235
	124	0,275349
	235	0,274615
	265	0,273151
	152	0,27064
	202	0,260268
	248	0,258509
	146	0,254382
	289	0,249229
	126	0,247459
	93	0,245609
	81	0,245373
	166	0,244903
	275	0,244825
	232	0,244668
	116	0,243809
	180	0,243809
	271	0,243809
	286	0,242795
	159	0,24264
	145	0,234474
	277	0,233316
	192	0,233008
	131	0,232547
	188	0,231933
	149	0,230937
	42	0,221178
	257	0,220951
	134	0,220575
	246	0,2205
	244	0,219748
	52	0,209862
	142	0,209789
	90	0,209604
	128	0,209604
	269	0,209567
	137	0,209531

288	0,213794	111	0,294932	272	0,418371	143	0,001053	276	0,276221	53	0,20942
289	0,213794	112	0,294932	51	0,395308	121	0,000968	118	0,274996	236	0,208795
111	0,213475	113	0,294932	240	0,395308	122	0,000968	52	0,271346	272	0,207987
202	0,213475	114	0,294932	195	0,394937	128	0,000968	248	0,26893	123	0,207544
209	0,213475	115	0,294932	255	0,394752	140	0,000968	202	0,258687	240	0,198952
243	0,213475	117	0,294932	43	0,394659	144	0,000968	146	0,250062	195	0,198808
259	0,213475	118	0,294932	123	0,389054	176	0,000968	122	0,24432	255	0,198736
263	0,213475	120	0,294932	126	0,370348	236	0,000968	209	0,24342	43	0,190344
265	0,213475	122	0,294932	139	0,370073	272	0,000968	143	0,242948	139	0,189206
188	0,213092	124	0,294932	289	0,369797	288	0,000968	288	0,242948	76	0,189076
192	0,213092	128	0,294932	86	0,369522	111	0,000967	219	0,242351	86	0,188995
219	0,213092	131	0,294932	105	0,369522	202	0,000967	142	0,241787	105	0,188995
261	0,213092	134	0,294932	254	0,369247	209	0,000967	212	0,236674	254	0,188889
48	0,212328	137	0,294932	184	0,36888	243	0,000967	232	0,236308	130	0,188784
110	0,212328	138	0,294932	279	0,368697	259	0,000967	90	0,234651	184	0,188749
146	0,212328	139	0,294932	204	0,368605	265	0,000967	155	0,231039	207	0,188713
269	0,212328	140	0,294932	276	0,368605	188	0,000965	201	0,226334	279	0,188678
286	0,212328	141	0,294932	118	0,368056	192	0,000965	242	0,226334	204	0,188643
50	0,21182	144	0,294932	206	0,359907	219	0,000965	187	0,226152	276	0,188643
141	0,21182	145	0,294932	142	0,353844	48	0,000963	200	0,225608	118	0,188432
203	0,21182	146	0,294932	143	0,345393	110	0,000963	286	0,224082	288	0,179779
212	0,210996	147	0,294932	280	0,345393	146	0,000963	53	0,222232	46	0,179676
218	0,210743	148	0,294932	281	0,345393	269	0,000963	45	0,213729	201	0,179676
248	0,210743	149	0,294932	288	0,345393	286	0,000963	240	0,212033	214	0,179676
232	0,210427	150	0,294932	201	0,345122	50	0,000961	43	0,204199	242	0,179676
123	0,199022	153	0,294932	214	0,345122	141	0,000961	192	0,203054	262	0,179676
167	0,19161	156	0,294932	242	0,345122	167	0,000961	188	0,201286	122	0,179641
121	0,188739	159	0,294932	262	0,345122	203	0,000961	76	0,200458	187	0,179641
45	0,187783	160	0,294932	122	0,345032	207	0,000961	244	0,192739	209	0,179641
136	0,176097	166	0,294932	187	0,345032	212	0,000959	289	0,19116	212	0,179641
47	0,175315	169	0,294932	209	0,345032	218	0,000958	207	0,190985	219	0,179641
54	0,175315	170	0,294932	212	0,345032	248	0,000958	126	0,190562	109	0,179607
82	0,175315	172	0,294932	219	0,345032	232	0,000957	261	0,185641	154	0,179607
112	0,175315	173	0,294932	109	0,344941	45	0,000921	47	0,183079	120	0,179538
113	0,175315	174	0,294932	88	0,344851	129	0,000908	54	0,183079	200	0,179538
114	0,175315	175	0,294932	120	0,34476	123	0,000886	82	0,183079	39	0,171024
115	0,175315	176	0,294932	200	0,34476	155	0,000848	112	0,183079	40	0,171024
117	0,175315	177	0,294932	39	0,322316	47	0,000847	113	0,183079	47	0,171024
118	0,175315	178	0,294932	40	0,322316	54	0,000847	114	0,183079	48	0,171024
124	0,175315	179	0,294932	47	0,322316	82	0,000847	115	0,183079	50	0,171024
132	0,175315	180	0,294932	48	0,322316	112	0,000847	117	0,183079	54	0,171024
134	0,175315	181	0,294932	50	0,322316	113	0,000847	138	0,183079	55	0,171024
135	0,175315	182	0,294932	54	0,322316	114	0,000847	147	0,183079	82	0,171024
138	0,175315	184	0,294932	82	0,322316	115	0,000847	153	0,183079	83	0,171024
139	0,175315	185	0,294932	83	0,322316	117	0,000847	160	0,183079	89	0,171024
147	0,175315	186	0,294932	89	0,322316	118	0,000847	168	0,183079	108	0,171024
153	0,175315	187	0,294932	108	0,322316	124	0,000847	169	0,183079	110	0,171024
160	0,175315	188	0,294932	110	0,322316	134	0,000847	171	0,183079	112	0,171024
169	0,175315	189	0,294932	112	0,322316	138	0,000847	172	0,183079	113	0,171024
172	0,175315	190	0,294932	113	0,322316	139	0,000847	173	0,183079	114	0,171024
173	0,175315	191	0,294932	114	0,322316	147	0,000847	174	0,183079	115	0,171024
174	0,175315	192	0,294932	115	0,322316	153	0,000847	175	0,183079	117	0,171024
175	0,175315	194	0,294932	117	0,322316	160	0,000847	177	0,183079	121	0,171024
177	0,175315	195	0,294932	138	0,322316	168	0,000847	178	0,183079	138	0,171024
178	0,175315	196	0,294932	141	0,322316	169	0,000847	179	0,183079	141	0,171024
179	0,175315	197	0,294932	144	0,322316	171	0,000847	181	0,183079	144	0,171024
180	0,175315	198	0,294932	147	0,322316	172	0,000847	182	0,183079	147	0,171024
181	0,175315	199	0,294932	148	0,322316	173	0,000847	185	0,183079	148	0,171024
182	0,175315	200	0,294932	150	0,322316	174	0,000847	186	0,183079	150	0,171024
184	0,175315	201	0,294932	153	0,322316	175	0,000847	189	0,183079	153	0,171024
185	0,175315	202	0,294932	160	0,322316	177	0,000847	190	0,183079	160	0,171024
186	0,175315	203	0,294932	169	0,322316	178	0,000847	191	0,183079	167	0,171024
187	0,175315	204	0,294932	170	0,322316	179	0,000847	193	0,183079	168	0,171024

189	0,175315	208	0,294932	172	0,322316	180	0,000847	194	0,183079	169	0,171024
190	0,175315	209	0,294932	173	0,322316	181	0,000847	196	0,183079	170	0,171024
191	0,175315	210	0,294932	174	0,322316	182	0,000847	197	0,183079	171	0,171024
194	0,175315	211	0,294932	175	0,322316	184	0,000847	198	0,183079	172	0,171024
195	0,175315	212	0,294932	176	0,322316	185	0,000847	199	0,183079	173	0,171024
196	0,175315	213	0,294932	177	0,322316	186	0,000847	208	0,183079	174	0,171024
197	0,175315	214	0,294932	178	0,322316	187	0,000847	210	0,183079	175	0,171024
198	0,175315	215	0,294932	179	0,322316	189	0,000847	211	0,183079	176	0,171024
199	0,175315	216	0,294932	181	0,322316	190	0,000847	213	0,183079	177	0,171024
200	0,175315	217	0,294932	182	0,322316	191	0,000847	215	0,183079	178	0,171024
201	0,175315	218	0,294932	185	0,322316	193	0,000847	216	0,183079	179	0,171024
204	0,175315	219	0,294932	186	0,322316	194	0,000847	217	0,183079	181	0,171024
208	0,175315	230	0,294932	189	0,322316	195	0,000847	230	0,183079	182	0,171024
210	0,175315	231	0,294932	190	0,322316	196	0,000847	231	0,183079	185	0,171024
211	0,175315	232	0,294932	191	0,322316	197	0,000847	234	0,183079	186	0,171024
213	0,175315	233	0,294932	194	0,322316	198	0,000847	239	0,183079	189	0,171024
215	0,175315	234	0,294932	196	0,322316	199	0,000847	241	0,183079	190	0,171024
216	0,175315	236	0,294932	197	0,322316	200	0,000847	245	0,183079	191	0,171024
217	0,175315	237	0,294932	198	0,322316	201	0,000847	249	0,183079	193	0,171024
229	0,175315	239	0,294932	199	0,322316	204	0,000847	250	0,183079	194	0,171024
230	0,175315	240	0,294932	203	0,322316	208	0,000847	252	0,183079	196	0,171024
231	0,175315	241	0,294932	208	0,322316	210	0,000847	264	0,183079	197	0,171024
234	0,175315	242	0,294932	210	0,322316	211	0,000847	266	0,183079	198	0,171024
239	0,175315	243	0,294932	211	0,322316	213	0,000847	267	0,183079	199	0,171024
241	0,175315	244	0,294932	213	0,322316	215	0,000847	268	0,183079	203	0,171024
242	0,175315	245	0,294932	215	0,322316	216	0,000847	274	0,183079	208	0,171024
245	0,175315	246	0,294932	216	0,322316	217	0,000847	278	0,183079	210	0,171024
246	0,175315	247	0,294932	217	0,322316	230	0,000847	282	0,183079	211	0,171024
249	0,175315	248	0,294932	218	0,322316	231	0,000847	283	0,183079	213	0,171024
250	0,175315	249	0,294932	230	0,322316	234	0,000847	284	0,183079	215	0,171024
251	0,175315	250	0,294932	231	0,322316	239	0,000847	285	0,183079	216	0,171024
252	0,175315	251	0,294932	234	0,322316	241	0,000847	287	0,183079	217	0,171024
254	0,175315	252	0,294932	239	0,322316	242	0,000847	95	0,181443	218	0,171024
255	0,175315	253	0,294932	241	0,322316	245	0,000847	269	0,177369	230	0,171024
257	0,175315	254	0,294932	245	0,322316	246	0,000847	128	0,17523	231	0,171024
264	0,175315	255	0,294932	249	0,322316	249	0,000847	236	0,174897	234	0,171024
266	0,175315	257	0,294932	250	0,322316	250	0,000847	272	0,174614	239	0,171024
267	0,175315	259	0,294932	252	0,322316	251	0,000847	137	0,164419	241	0,171024
268	0,175315	260	0,294932	253	0,322316	252	0,000847	149	0,159407	245	0,171024
274	0,175315	262	0,294932	259	0,322316	254	0,000847	237	0,15702	249	0,171024
276	0,175315	264	0,294932	260	0,322316	255	0,000847	44	0,154527	250	0,171024
277	0,175315	265	0,294932	263	0,322316	257	0,000847	127	0,153952	252	0,171024
278	0,175315	266	0,294932	264	0,322316	264	0,000847	79	0,152904	253	0,171024
279	0,175315	267	0,294932	266	0,322316	266	0,000847	84	0,147014	259	0,171024
282	0,175315	268	0,294932	267	0,322316	267	0,000847	92	0,146946	260	0,171024
283	0,175315	269	0,294932	268	0,322316	268	0,000847	42	0,145361	264	0,171024
284	0,175315	271	0,294932	274	0,322316	274	0,000847	136	0,14263	266	0,171024
285	0,175315	272	0,294932	278	0,322316	276	0,000847	103	0,138852	267	0,171024
287	0,175315	273	0,294932	282	0,322316	277	0,000847	123	0,136385	268	0,171024
58	0,165072	274	0,294932	283	0,322316	278	0,000847	131	0,135851	274	0,171024
94	0,157571	276	0,294932	284	0,322316	279	0,000847	145	0,134523	278	0,171024
76	0,152041	277	0,294932	285	0,322316	282	0,000847	159	0,133173	282	0,171024
130	0,151742	278	0,294932	287	0,322316	283	0,000847	275	0,132516	283	0,171024
157	0,150558	279	0,294932	58	0,31453	284	0,000847	152	0,132301	284	0,171024
275	0,15048	282	0,294932	94	0,303708	285	0,000847	125	0,13074	285	0,171024
125	0,149547	283	0,294932	103	0,300494	287	0,000847	166	0,130371	287	0,171024
127	0,148561	284	0,294932	76	0,286607	206	0,000835	116	0,129977	135	0,153064
154	0,148561	285	0,294932	129	0,253644	136	0,000788	183	0,129725	84	0,131911
206	0,148095	286	0,294932	84	0,233475	76	0,000726	271	0,129338	261	0,131291
84	0,147763	287	0,294932	238	0,228825	92	0,000721	102	0,129249	79	0,115185
129	0,14769	288	0,294932	155	0,228505	43	0,000719	133	0,127265	125	0,106489
116	0,146741	129	0,224607	125	0,214892	157	0,000712	81	0,126182	133	0,104936
205	0,146722	155	0,220671	275	0,213465	281	0,000703	93	0,121644	183	0,104706

155	0,146274	58	0,210237	133	0,210477	125	0,000699	247	0,120186	96	0,096743
152	0,142616	206	0,204231	235	0,210476	183	0,000694	96	0,111076	229	0,095975
235	0,141237	125	0,173419	116	0,209915	84	0,00069	56	0,104825	270	0,095827
238	0,14105	76	0,173307	127	0,203144	280	0,00069	85	0,103468	136	0,091807
103	0,138435	157	0,172934	96	0,188882	56	0,000576	238	0,10323	56	0,091399
133	0,136455	103	0,171813	136	0,179197	49	0,000568	235	0,102562	92	0,088644
46	0,135615	84	0,170884	152	0,172843	44	0,000559	41	0,099597	103	0,086084
49	0,112362	133	0,168844	256	0,153935	94	0,000559	94	0,095709	49	0,084235
102	0,106606	49	0,151166	80	0,146081	88	0,000555	273	0,094877	91	0,083756
85	0,105806	94	0,146044	49	0,143316	103	0,000554	49	0,092677	94	0,082007
142	0,104428	96	0,13088	151	0,137024	41	0,000544	233	0,092548	132	0,078315
79	0,100976	258	0,130532	85	0,131491	133	0,000541	80	0,091756	258	0,077454
96	0,09838	41	0,129446	205	0,128918	79	0,000538	151	0,085973	41	0,077355
41	0,098119	270	0,127174	41	0,123431	96	0,000535	258	0,069675	44	0,076225
151	0,089829	79	0,1259	87	0,121826	102	0,000501	57	0,065687	45	0,061958
80	0,082675	87	0,105027	102	0,119847	258	0,000474	256	0,065222	155	0,05243
87	0,071126	102	0,103698	45	0,11959	95	0,000466	91	0,061946	102	0,048848
256	0,063573	136	0,073535	95	0,106629	51	0,00039	156	0,057387	87	0,044254
258	0,062011	123	0,0726	158	0,099052	58	0,000183	158	0,053882	129	0,040998
55	0,056312	45	0,0711	258	0,072023	87	0,000182	87	0,045074	95	0,037151
158	0,05373	95	0,054238	270	0,059348	91	0,000117	58	0,037788	58	0,034665
