# Supplementary material for: Insights into the evolution of sorbitol metabolism: phylogenetic analysis of SDR196C family
Source: BMC Evol Biol. 2012 Aug 16;12:147. doi: 10.1186/1471-2148-12-147 (PMC3458964; doi:10.1186/1471-2148-12-147)
Supplement: Additional file 10 — Candidates for amino acid sites related with functional divergence. The numbering used corresponds with that of the alignment implemented by DIVERGE. Cat. I refers to Category I and Cat. II refers to Category II. A: Cat. II; conserved tandem in Lineage II and variable in Linage I. B: Cat. I; Conserved tandem in Lineage I and variable in Lineage V and Cat. II; conserved tandem in Lineage V and variable in Lineage I. C: Cat. I; conserved tandem in Lineage V and variable in Lineage VI. D: Cat. I; conserved tandem in Lineage I and variable in Lineage VI and Cat. II; conserved tandem in Lineage VI and variable in Lineage I. E: Cat. I; conserved tandem in Lineage II and variable in Lineage VI and Cat. II; conserved tandem in Lineage VI and variable in Lineage II. Critical amino acids responsible for functional divergence are shown in the R. sphaeroides SDH crystal structure (pdb:1k2w). Divergent residues of category I are depicted in red and those of category II are depicted in blue. [file 1471-2148-12-147-S10.pdf]

A

Lineages I-II

Lineage	Species	112<br>697<br>830
I	B.mallei	VLA
	B.ambifaria	VIA
	B.cenocepacia	VIA
	Burkholderiasp.1	VIS
	Burkholderiasp.2	ILA
	Burkholderiasp.3	ILA
	B.multivorans	IIA
	R.solanaceum	IIT
	B.phymatum	VLS
	B.phytofirmans	ILA
	B.thailandensis	VLA
	B.vietnamensis	IIA
	B.xenovorans	ILA
	B.dolosa	IIA
	B.graminis	VLA
	Pseudomonas	VIA
	B.glumae	VLA
II	A.avenae	IID
	V.paradoxus	IID
	V.eiseniae	IID
	Marinomonas	IID
	R.bacterium	IID
	Thalassiobium	IID

Cat. II

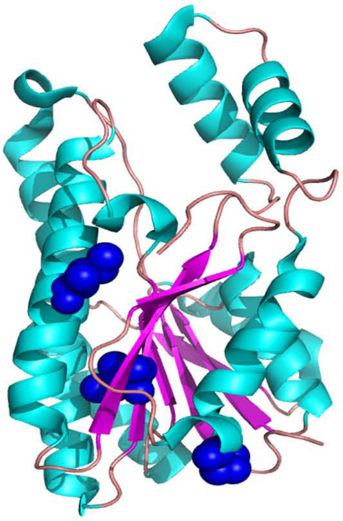

B

Lineages I-V

Lineage	Species	9<br>1	Species	1<br>96<br>08
I	B.mallei	R	B.mallei	AR
	B.ambifaria	R	B.ambifaria	ER
	B.cenocepacia	R	B.cenocepacia	TR
	Burkholderiasp.1	R	Burkholderiasp.1	ER
	Burkholderiasp.2	R	Burkholderiasp.2	ER
	Burkholderiasp.3	R	Burkholderiasp.3	EH
	B.multivorans	R	B.multivorans	ER
	R.solanaceum	R	R.solanaceum	ER
	B.phymatum	R	B.phymatum	GH
	B.phytofirmans	R	B.phytofirmans	QC
	B.thailandensis	R	B.thailandensis	AR
	B.vietnamensis	R	B.vietnamensis	ER
	B.xenovorans	R	B.xenovorans	ER
	B.dolosa	R	B.dolosa	AR
	B.graminis	R	B.graminis	ER
	Pseudomonas	R	Pseudomonas	TR
	B.glumae	R	B.glumae	AR
V	A.tumefaciens	A	A.tumefaciens	DR
	R.etli	T	R.etli	DR
	O.intermedium	D	O.intermedium	DR
	O.anthropi	K	O.anthropi	DR

Cat. I **Cat. II**

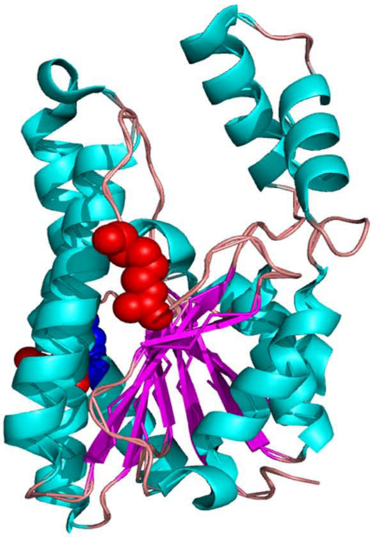

C

Lineages V-VI

Lineage	Species	8<br>0
V	A.tumefaciens	K
	R.etli	K
	O.intermedium	K
	O.anthropi	K
VI	R.sphaeroides	G
	Citreicella	Q
	P.bermudensis	E
	Jannaschia	Q
	H.phototrophica	E
	R.bacteriumKLH11	E
	Roseobacter	D
	L.vestfoldensis	H
	S.lacuscaerulensis	E
	L.alexandrii	E
	Roseibium	E
	P.marinum	E
	P.gallaeciensis	K
	Silicibacter	E
	M.alkaliphilus	E
	R.capsulatus	H
	P.denitrificans	Q
	Ahrensia	H
	O.antarcticus	Q
	S.stellata	H
	Azospirillum	A
	L.aggregata	R
	P.savastanoi	S
	P.syringae	S
	P.fluorescens	A
	Rhodobacter	H
	A.radiobacter	Q
	M.opportunistum	K
	R.loti	K
	A.vitis	H
	Mesorhizobium	E
	Rhizobium	E
	S.medicae	E
	R.meliloti	Q
	S.meliloti	Q

Cat. I

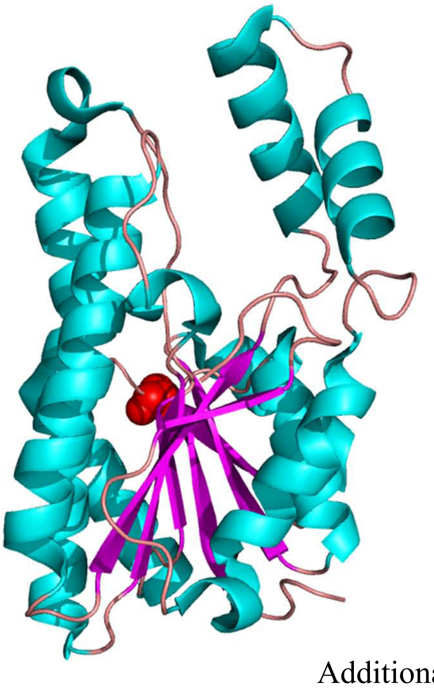

Additional File 10. Sola-Carvajal *et al*. 2012.
