# Supplementary material for: Insights into the evolution of sorbitol metabolism: phylogenetic analysis of SDR196C family
Source: BMC Evol Biol. 2012 Aug 16;12:147. doi: 10.1186/1471-2148-12-147 (PMC3458964; doi:10.1186/1471-2148-12-147)
Supplement: Additional file 4 — Multiple sequence alignments (MSAs) obtained with GUIDANCE and their corresponding Guidance scores [[25,26]. The file shows the result obtained for the MSA of the 6 lineages, 3 sublineages and 16S rRNA. In all cases, an overall quality assessment (Guidance score) of above 0.97 was obtained with the MSA algorithm PRANK [27]. [file 1471-2148-12-147-S4.pdf]

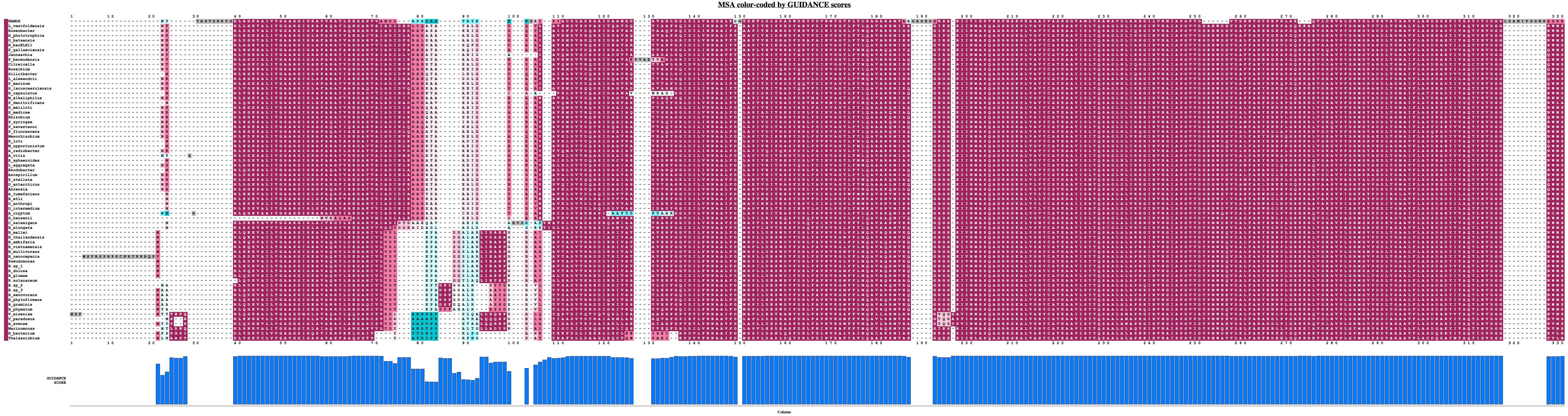

Legend:

The alignment confidence scale:

Confident <---> Uncertain

Insufficient Data

MSA of SDR196_C Family - GUIDANCE alignment score: 0.974472

Additional File 4. Sola-Carvajal *et al.* 2012..

MSA color-coded by GUIDANCE scores

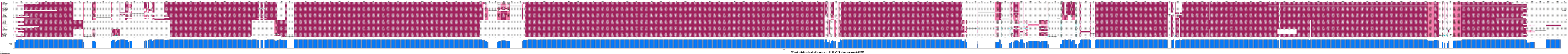

MSA of 16S rRNA (nucleotide sequence) - GUIDANCE alignment score: 0.986227

Additional File 4. Sola-Carvajal *et al*. 2012.

MSA color-coded by GUIDANCE scores

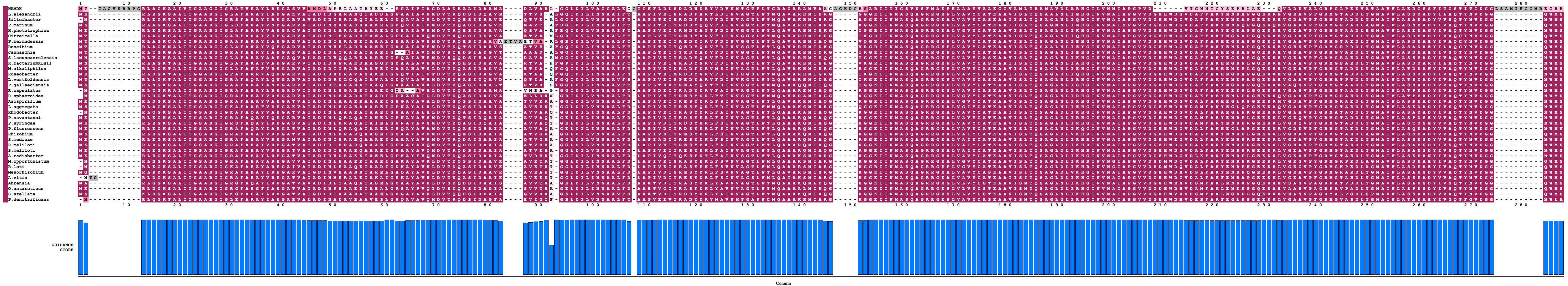

Legend:

The alignment confidence scale:

9 8 7 6 5 4 3 2 1

Confident <---> Uncertain

Insufficient Data

MSA of Linage VI - GUIDANCE alignment score: 0.994302

Additional File 4. Sola-Carvajal *et al*. 2012.
